# Biologically Enhanced Genome-Wide Association Study Provides Further Evidence for Candidate Loci and Discovers Novel Loci That Influence Risk of Anterior Cruciate Ligament Rupture in a Dog Model

**DOI:** 10.3389/fgene.2021.593515

**Published:** 2021-03-05

**Authors:** Lauren A. Baker, Mehdi Momen, Rachel McNally, Mark E. Berres, Emily E. Binversie, Susannah J. Sample, Peter Muir

**Affiliations:** ^1^Department of Surgical Sciences, School of Veterinary Medicine, University of Wisconsin-Madison, Madison, WI, United States; ^2^Bioinformatics Resource Center, Biotechnology Center, University of Wisconsin-Madison, Madison, WI, United States

**Keywords:** ACL rupture, ACL, cruciate rupture, dog model, anterior cruciate ligament, cruciate ligament, GWAS, dog GWAS

## Abstract

Anterior cruciate ligament (ACL) rupture is a common condition that disproportionately affects young people, 50% of whom will develop knee osteoarthritis (OA) within 10 years of rupture. ACL rupture exhibits both hereditary and environmental risk factors, but the genetic basis of the disease remains unexplained. Spontaneous ACL rupture in the dog has a similar disease presentation and progression, making it a valuable genomic model for ACL rupture. We leveraged the dog model with Bayesian mixture model (BMM) analysis (BayesRC) to identify novel and relevant genetic variants associated with ACL rupture. We performed RNA sequencing of ACL and synovial tissue and assigned single nucleotide polymorphisms (SNPs) within differentially expressed genes to biological prior classes. SNPs with the largest effects were on chromosomes 3, 5, 7, 9, and 24. Selection signature analysis identified several regions under selection in ACL rupture cases compared to controls. These selection signatures overlapped with genome-wide associations with ACL rupture as well as morphological traits. Notable findings include differentially expressed *ACSF3* with *MC1R* (coat color) and an association on chromosome 7 that overlaps the boundaries of *SMAD2* (weight and body size). Smaller effect associations were within or near genes associated with regulation of the actin cytoskeleton and the extracellular matrix, including several collagen genes. The results of the current analysis are consistent with previous work published by our laboratory and others, and also highlight new genes in biological pathways that have not previously been associated with ACL rupture. The genetic associations identified in this study mirror those found in human beings, which lays the groundwork for development of disease-modifying therapies for both species.

## Introduction

The anterior cruciate ligament (ACL) is a ligament spanning from the lateral femoral condyle to the proximal tibia that provides crucial stability to the knee joint, counteracting anterior translation, hyperextension, and internal rotation of the tibia (Noyes, 2009). ACL rupture is most often a midsubstance failure of this ligament (van der List et al., 2017), which occurs for multiple and complex reasons including genetic predisposition (Smith et al., 2012a,b). Standard of care includes physical therapy alone or after surgical reconstruction. Unfortunately, neither treatment prevents the long-term development of post-traumatic osteoarthritis (OA) (Lohmander et al., 2007) and disease-modifying therapies are critically needed. The key to disease-modifying therapy may lie within the underlying genetic predisposition to ACL rupture. Multiple studies have been performed in search of genetic drivers of disease, but discoveries have been limited, mostly due to inadequate sample composition (e.g., male-only samples) and size (John et al., 2016).

Anterior cruciate ligament rupture in the dog is a useful genomic model for human ACL rupture. The onset and progression of the condition is remarkably similar between humans and dogs (Baker et al., 2018). There are several advantages to the dog as a genomic model for ACL rupture that have been discussed previously (Baker et al., 2017, 2018), including higher disease prevalence (Witsberger et al., 2008), established heritability of the disease (Nielen et al., 2003; Wilke et al., 2006; Baker et al., 2017), and within breed homogeneity and extensive linkage disequilibrium (LD) (Karlsson and Lindblad-Toh, 2008). While genome-wide association studies (GWAS) have been performed (Wilke et al., 2009; Baird et al., 2014a,b; Hayward et al., 2016, 2019; Baker et al., 2017, 2018; Huang et al., 2017), the associations identified have not been repeatable from one study to the next. Our previous work on the genetic basis of ACL rupture in the Labrador Retriever (Baker et al., 2017, 2018) supports the hypothesis that ACL rupture is highly polygenic, and that most, if not all, single nucleotide polymorphisms (SNP) effects are relatively small. While we have identified some reasonable candidate genes, the majority of the identified associations do not have clear relevance to ACL rupture.

In the present study, we embrace the polygenicity of ACL rupture with a Bayesian approach to GWAS. In contrast to traditional single-marker models [e.g., linear mixed models (LMM)], Bayesian models for GWAS estimate the combined effect of all SNPs in the dataset. A Bayesian approach could treat all SNP associations as random effects drawn from a normal distribution which allows for an unbiased estimate of variance explained by the SNPs (Moser et al., 2015). This approach can be tailored further to GWAS of complex phenotypes by treating SNP effects as drawn from a mixture of normal distributions corresponding to differing SNP effect sizes, including a distribution for SNPs with zero effect. BayesR (Erbe et al., 2012; Moser et al., 2015) is one such implementation that models SNP effects using four normal distributions with variance ranging from zero to 1% of total genetic variance, which more accurately models the effect size distribution expected from a complex phenotype. BayesR has been shown to be equal or superior to the LMM for both prediction modeling and QTL mapping (Moser et al., 2015; Kemper et al., 2015).

Another advantage of the Bayesian approach to GWAS is the ease with which prior biological information can be incorporated into the model (Stephens and Balding, 2009). Most single marker models, including the LMM, assume each SNP has an equal probability of having an effect on the phenotype of interest. However, SNPs that are within or near candidate genes may have a higher probability of being associated with the phenotype. Bayesian models allow the user to set a higher prior probability of effect to these SNPs. While there is some subjectivity to assigning prior probabilities, this is an improvement from the arguably arbitrary way biological knowledge is used to interpret results after GWAS analysis (Stephens and Balding, 2009; MacLeod et al., 2016; Gallagher and Chen-Plotkin, 2018). BayesRC (MacLeod et al., 2016) is a modification to BayesR that can incorporate prior biological knowledge as a part of the analysis. To do this, SNPs are assigned to separate classes, defined by the user, based on whether the classes differ in the prior likelihood that they contain variants that are associated with the phenotype. This method improves the power and precision to detect associated variants when compared to BayesR (MacLeod et al., 2016).

The purpose of this study was to incorporate knowledge of ACL rupture candidate genes with BayesRC GWAS to identify and prioritize genetic variants with clear relevance to the disease process and evaluate the repeatability of associations previously reported in the literature. We defined candidate genes through differential gene expression analysis of RNA sequencing data and published literature. SNPs within candidate genes were assigned to biological priors. We discovered associations in genes from molecular pathways that were not previously implicated in ACL rupture pathogenesis and replicated associations that have been previously reported in studies of ACL rupture in both human beings and dogs. Many of these associations are within haplotypes that are under selection in the Labrador Retriever.

## Materials and Methods

### Data Collection and Phenotyping

All procedures were performed in accordance with the recommendations in the Guide for the Care and Use of Laboratory Animals of the National Institutes of Health and the American Veterinary Medical Association and with approval from the Animal Care Committee of the University of Wisconsin-Madison (protocols V1070 and V5463). Informed consent of each owner was obtained before participation in the study. Recruitment and quality control have been reported in previous publications by our laboratory (Baker et al., 2017, 2018). Client-owned Labrador Retriever dogs (*Canis lupus familiaris*) were recruited from the University of Wisconsin-Madison UW Veterinary Care teaching hospital, online advertising, and through local and national breed clubs. If available, a pedigree was collected from each dog to confirm purebred status. A single ACL rupture was sufficient to consider a dog a case. All cases were diagnosed by a veterinarian. In the vast majority of cases, a ruptured ACL was confirmed during knee stabilization surgery. Control dogs were over the age of 8 years (Reif and Probst, 2003) with a normal orthopaedic exam and knee radiographs with no evidence of ACL rupture (joint effusion or osteophytosis) (Chuang et al., 2014). Age, weight, and whether the dog was spayed or neutered were recorded. DNA was extracted from blood or saliva. Dogs were genotyped using the Illumina Canine HD BeadChip (220,000 SNPs).

### Imputation

Dogs genotyped on the Illumina HD BeadChip were imputed to the higher density Thermo Fisher Scientific Axiom Canine HD array (710,000 SNPs) using Beagle 5.0 (Browning et al., 2018) and a previously described method (Friedenberg and Meurs, 2016). Our reference panel consisted of *N* = 646 dogs that were genotyped on a pre-commercial version of the Axiom array, including 96 Labrador Retrievers. These data were obtained from the laboratory of Dr. Brian Davis of Texas A&M University and are the subject of an unpublished research project and are therefore not currently available to the public. To validate the imputation method in our population, we used whole-genome sequencing (WGS) data from *n* = 22 Labrador Retrievers that were sequenced for an unrelated project in our laboratory. 173,662 SNPs, present on the Illumina Canine HD BeadChip, were extracted from WGS data to create a test set for the imputation method. The test dataset was imputed with the multibreed reference, a window size of 3 cm with a 1 cm overlap, and effective population size of 100. The effective population size of the Labrador Retriever was based on results from two studies, one that states the effective population size is 114 (Calboli et al., 2008), and another more recent study that states the effective population size is 82 (Wiener et al., 2017). To evaluate accuracy, bi-allelic genotypes at imputed SNPs from the 22 Labrador Retrievers with WGS data were compared to the genotypes at the same SNP locations extracted from WGS data. If the complete imputed genotype matched the complete WGS genotype, the SNP genotype was scored as correct. Accuracy of imputation was calculated per chromosome as number of genotypes imputed correctly divided by the number of genotypes compared. Overall, accuracy exceeded 90% for all autosomes, and the vast majority of autosomes (36/38) achieved accuracy of 96% or higher (Supplementary Table 1). Given these acceptable results, we moved forward with imputation of our study dataset using Beagle 5.0 and the aforementioned parameters.

Our final dataset included 397 (156 ACL rupture affected and 241 unaffected control) purebred Labrador Retriever dogs. Of these, 55 were intact males, 30 were intact females, 161 were castrated males, and 151 were ovariohysterectomized females. A total of 237 dogs were part of a previously published GWAS analysis (Baker et al., 2017). Quality control on the imputed data was performed using PLINK v1.9 (Chang et al., 2015). SNPs were removed from the dataset if they had minor allele frequency (MAF) < 0.01, genotyping call rate <90%, or did not conform to Hardy–Weinberg proportions at a *P*-value less than 1E-07. Because BayesRC does not tolerate missing genotypes, SNPs with any missing genotypes were also removed from the dataset. After quality control 443,227 SNPs remained for analysis.

### RNA Sequencing and Differential Gene Expression Analysis

Anterior cruciate ligament and knee synovial tissue biopsies were collected from four ACL rupture affected cases and four unaffected control dogs. These dogs were recruited under the same phenotyping criteria that was established for genotyping. It was important to examine both ACL and synovium, as synovitis is known to precede ACL rupture in the dog (Bleedorn et al., 2011) and may play a role in disease progression and development of OA (Comerford et al., 2011). Cases and controls were matched as closely as possible based on breed, sex, neutered status, age, and weight. Medications that the dogs were taking at the time of sample collection were also considered. We prioritized sample size and quality above all other variables, therefore, two matched pairs of Golden Retrievers were chosen with two matched pairs of Labrador Retrievers for this analysis (Table 1). In phylogenetic terms, the Golden Retriever is closely related to the Labrador Retriever (Parker et al., 2017). Tissues from cases were collected during knee stabilization surgery. Tissues from unaffected control dogs were collected from dogs undergoing pelvic limb amputation or euthanasia for reasons unrelated to this study. Library preparation and sequencing was performed at the University of Wisconsin-Madison Biotechnology Center (Madison, WI, United States). Illumina TruSeq RNA libraries were constructed and 150 bp paired-end sequencing was performed using the Illumina Hi-Seq 2500 platform. Read quality was evaluated using FastQC (Andrews, 2010).

**TABLE 1:** Breed, sex, age, and weight of matched case and control pairs chosen for RNA sequencing analysis.

**Cases**				**Matched controls**			
**Breed**	**Sex**	**Age (year)**	**Weight (kg)**	**Breed**	**Sex**	**Age (year)**	**Weight (kg)**
GR	CM	8.8	30.5	GR	CM	14.9	N/A
GR	CM	5.6	44.0	GR	CM	3.9	34.0
LR	CM	9.7	36.0	LR	CM	12.7	28.5
LR	CM	13.3	36.0	LR	CM	13.5	35.0

*GR, Golden Retriever; LR, Labrador Retriever; CM, castrated male. Weight at the time of death was not available for one dog.*

Bioinformatic analysis of RNASeq reads adhered to ENCODE guidelines and best practices for RNASeq (Encode Consortium,, 2016). Briefly, alignment of adapter-trimmed (Skewer v0.1.123) (Jiang et al., 2014) 2 × 150 bp paired-end strand-specific Illumina reads to the canFam3.1 genome (assembly accession: GCA_000002285.2) was achieved with the Spliced Transcripts Alignment to a Reference (STAR v2.5.3a) software (Dobin et al., 2013), and a splice-junction aware aligner using Ensembl annotation (Aken et al., 2016). Expression estimation was conducted using RSEM v1.3.0 (RNASeq by Expectation Maximization) (Li and Dewey, 2011). To test for differential expression among individual group contrasts, expected read counts were used as input into edgeR v3.16.5 (Robinson et al., 2010). Significance of the negative-binomial test was adjusted with a Benjamini–Hochberg false discovery rate (FDR) correction at the 5% level (Reiner et al., 2003). Before statistical analysis with edgeR, independent filtering was performed, requiring a threshold of at least 1 read per million in two or more samples. The validity of the Benjamini–Hochberg FDR multiple testing procedure was evaluated by inspection of the uncorrected *P*-value distribution. Lists of differentially expressed genes (DEGs) were submitted for pathway analysis using the PANTHER classification system v15.0 (Mi et al., 2013; Mi et al., 2019) to analyze for statistical overrepresentation using the Fisher’s Exact test. Significance was defined as *P* < 0.05 after correction for FDR.

### Association Analysis and Assignment of Biological Priors

We used the BayesRC algorithm (MacLeod et al., 2016) to perform a genome-wide association analysis that incorporated prior biological knowledge. A copy of the software was obtained electronically via Dr. Iona MacLeod (MacLeod et al., 2016). BayesRC is an extension of the Bayesian mixture model (BMM) BayesR (Erbe et al., 2012; Moser et al., 2015). The BayesR algorithm assumes that SNP effects are derived from a mixture of four normal distributions including a zero-effect distribution. The three effect distributions are *N*(0, $0.0001\,\sigma_{\text{g}}^{2}$), *N*(0, $0.001\,\sigma_{\text{g}}^{2}$), and *N*(0, $0.01\,\sigma_{\text{g}}^{2}$), with $\sigma_{\text{g}}^{2}$ representing the additive genetic variance explained by the SNPs. This mixture of distributions approximates the various SNP effect sizes that would typically describe the underlying genetic architecture of complex traits (Moser et al., 2015). A Markov Chain Monte Carlo (MCMC) approach is used to estimate SNP effects from the four distributions. As the algorithm runs, it uses the data to estimate the probability that each SNP belongs within distribution 1, 2, 3, and 4, and updates the proportions each iteration.

To incorporate biological information, BayesRC directs the user to assign each SNP to a class (of 2 or more classes) where each class represents some biological information. For example, SNPs within the boundaries of candidate genes could be assigned to one class, and all other SNPs would be assigned to separate class. SNPs that receive the same class assignment are analyzed together, and each class is analyzed separately from other classes. The BayesRC algorithm updates the distribution of SNP effects within each class and separate from other classes, which is an advantage if any one class is enriched for associated loci. A uniform prior is applied across all classes to ensure that biological information only influences the analysis if the data supports it (MacLeod et al., 2016). We used a mostly uninformative Dirichlet prior [α = (1,1,1,1)]across classes to define the prior proportion of SNPs in each distribution (MacLeod et al., 2016).

We defined a total of five biological prior classes using the results of our RNASeq analysis and peer-reviewed literature (Table 2 and Supplementary Figure 1). Three biological prior classes were defined using candidate genes identified through RNASeq and differential gene expression analysis: DEGs in ACL (“ACL”), DEGs in knee synovium (“SYN”), and DEGs identified in both tissues (“A&S”). A fourth class was defined using candidate genes that have been reported in peer-reviewed literature as associated with ACL rupture or tendinopathy in humans and/or dogs (“LIT,” Table 3). SNPs were assigned to a class if they were within the boundaries of a candidate gene ±25 kb. The size of the flanking region was conservatively defined by calculating the average haplotype block size in our data using PLINK, which was 19.43 kb with a maximum haplotype block size of 200 kb. Gene boundaries were based on canFam3.1 from Ensembl release 97 using the python package PyEnsembl v1.7.5.

**TABLE 2:** The number of SNPs assigned to biological priors defined by differential gene expression analysis and candidate genes reported in the literature.

**Class**	**Definition**	**Number of SNPs**
ACL	Differentially expressed genes in ACL	2,614
SYN	Differentially expressed genes in knee synovium	7,850
A&S	Differentially expressed genes in ACL and knee synovium	703
LIT	Gene associations reported in peer-reviewed literature	1,042
NA	SNPs not assigned to biological priors	431,018

*SNP, single nucleotide polymorphism.*

**TABLE 3:** Candidate genes for ACL rupture derived from peer-reviewed literature in humans and dogs.

**Gene**	**Human**	**Dog**
*ACAN*	Mannion et al., 2014; Johnson et al., 2015	Wilke, 2010
*BGN*	Mannion et al., 2014	
*COL1A1*	Khoschnau et al., 2008; Posthumus et al., 2009a; Ficek et al., 2013; Stȩpień-Słodkowska et al., 2013	Baird et al., 2014a
*COL3A1*	O’Connell et al., 2015; Stȩpień-Słodkowska et al., 2015b	Baird et al., 2014a
*COL5A1*	Posthumus et al., 2009b; Stȩpień-Słodkowska et al., 2015a	Baird et al., 2014a
*COL5A2*		Baird et al., 2014a
*COL6A1*	O’Connell et al., 2015	
*COL9A1*		Wilke et al., 2005
*COL11A1*		Baird et al., 2014a
*COL11A2*	Saunders et al., 2016	
*COL12A1*	Posthumus et al., 2010; Ficek et al., 2013	
*COL24A1*		Baird et al., 2014a
*COLGALT1*	Kim et al., 2017	
*DCN*	Mannion et al., 2014	
*DIRC2*		Baird et al., 2014a
*ELN*	El Khoury et al., 2015	
*FBN1*		Baird et al., 2014a
*FBN2*	El Khoury et al., 2015	
*FMOD*	Mannion et al., 2014	
*GDF5*	Raleigh et al., 2013	
*HIF1A*	Rahim et al., 2014	
*IL1B*	Rahim et al., 2017	
*IL6*	Lulińska-Kuklik et al., 2019	
*ITGB3*	Saunders et al., 2016	
*KDR*	Rahim et al., 2014	
*LOX*	Saunders et al., 2016	Baird et al., 2014a
*LTBP2*		Baird et al., 2014a
*LUM*	Mannion et al., 2014	
*MMP1*	Posthumus et al., 2012	
*MMP3*	Collins and Raleigh, 2009; Posthumus et al., 2012	
*MMP10*	Posthumus et al., 2012	
*MMP12*	Posthumus et al., 2012	
*NGFB*	Rahim et al., 2014	
*RNF152*		Baird et al., 2014b
*SEMA5B*		Baird et al., 2014b
*SORCS2*		Baird et al., 2014b
*TNC*	Collins and Raleigh, 2009; Gibbon et al., 2018	
*VCAN*		Wilke, 2010
*VEGFA*	Rahim et al., 2014	
*WISP2*	Johnson et al., 2015	
*ZDHHC23*		Baird et al., 2014b

Because Labrador Retrievers in the current dataset were present in the datasets of our previously published work [Baker et al., 2017 (*N* = 237 dogs), 2018 (*N* = 222 dogs)], candidate genes identified through significant associations from our previous studies were not included in the peer-reviewed literature class to avoid introducing bias. These genes included *PPP1R16B* (Baker et al., 2017), *DOCK2* (Baker et al., 2018), and *ROR2* (Baker et al., 2018). We have previously reported a weak association with the gene *ACAN* (Baker et al., 2017). We chose to include *ACAN* as a part of our peer-reviewed literature class because our previously reported association did not meet genome-wide significance, and it is an especially interesting candidate gene for degenerative ligament disease that has been reported in human (Mannion et al., 2014; Johnson et al., 2015), horse (Plaas et al., 2011), and dog genetic research, including work that was independent of our research group (Wilke, 2010). SNPs that were not within or near candidate genes were assigned to a separate class. Ultimately, 12,209 SNPs were assigned to a biological prior.

We ran the BayesRC algorithm for a total of 200,000 iterations with a burn-in period of 100,000 iterations. The model analysis was repeated five times to assess model convergence. Fixed effects included in the analysis were dog sex, age, weight, and neuter status (Whitehair et al., 1993; Witsberger et al., 2008). To account for population structure in the dataset, the top five principal components derived from eigen decomposition of the variance-standardized genetic relationship matrix were also included as fixed effects in the model. Principal components analysis was performed using PLINK v1.9. Final mean SNP effects were evaluated based on the absolute value of the reported SNP effect. SNP effects were assigned to genes if they were within a gene boundary ±25 kb. For the purpose of comparing results with and without assignment of biological priors, we performed an analysis with all of the above parameters, but assigned all SNPs to a single prior class, which is effectively equivalent to a BayesR analysis.

### Selection Signature Analysis

Anterior cruciate ligament rupture in dogs has a marked breed predisposition, with reported breed prevalence in the Labrador Retriever of 5.79% (Witsberger et al., 2008). Artificial selection is a necessary part of breed creation, and genetic risk of ACL rupture in the Labrador Retriever may be the result of unintentional selection due to linkage between ACL rupture risk variants and desirable traits. Regions of the genome that have been under selection have reduced heterozygosity which is identifiable through selection signature analysis. ACL rupture risk variants that are also within regions of the genome that are under selection may be especially important to defining breed predisposition to ACL rupture. We performed selection signature analysis to detect regions that show preferential selection in the genomes of case versus control subpopulations. We performed whole genome scans for signatures of selection based on the concept of extended haplotype homozygosity (EHH) as formulated by Sabeti et al. (2002). In EHH analysis, reduction in haplotype diversity is computed as the probability that two extended haplotypes around a given locus are the same, given that they have the same allele at the locus.

We defined haplotypes for case and control subpopulations using fastPHASE software (Scheet and Stephens, 2006) with the number of random starts set to 10 (-T10) and the number of iterations set to 20 (-C20). The fastPHASE model is based on the idea that, over short regions of the genome, haplotypes in a population tend to cluster into groups of similar haplotypes. The number of clusters, *K*, is an essential hyperparameter that must be computed. To define *K*, a portion of the data is set to missing, and for several values of *K*, fastPHASE makes a best guess for the missing genotypes. This process is repeated multiple times, each time choosing a different portion of the observed data to set to missing. The chosen value for *K* is the one that produced the lowest overall error rate. We assigned the upper limit for the number of clusters equal to 40 (-Ku40) and the lower limit to 10 (-Kl10), with an interval of 5 (-Ki5). The masking procedure was repeated 100 times (-Ks100), randomly selecting 500 SNP loci (-Ks500), and 5% of observed genotypes among individuals (-Kp0.05) to be masked.

To define selection signatures, we calculated the cross-population extended haplotype heterozygosity test (XP-EHH) using the R package “rehh” v.3.1.0 (Gautier and Vitalis, 2012). XP-EHH compares the integrated EHH between two populations at the same SNP. Selection signatures are identified based on overrepresented haplotypes in one population compared to the other (Sabeti et al., 2007). We evaluated case and control populations to assess whether selection pressures have affected individuals in the case category relative to the founder population (unaffected control dogs) (Voight et al., 2006). Candidate SNPs were defined using a threshold of −log_10_(*P* ≤ 1E-05). Genome-wide significance was defined at −log_10_(*P* ≤ 1E-08). We used the “calc_haplen” function within “rehh” to define the length of the average haplotype around each significant marker, and each haplotype was evaluated for genes that may be driving selection using the canFam 3.1 gene annotation.

## Results

### RNA Sequencing

FastQC analysis determined that all samples were of good quality. Overall, average coverage and mapping were excellent across samples. There were 98,214,398 average reads per sample. The average primary and secondary alignment percentages were 90.18 and 8.21%, respectively. The average proportion of properly paired reads was 99.97%. After adjustment for multiple testing and without imposing a threshold for log fold change, we identified 200 genes from ACL tissue and 444 genes from synovium tissue that were significantly differentially expressed between case and control dogs (Supplementary Tables 2, 3). To ease interpretation of results, only transcript ID’s that could be matched to a known gene were included in the assignment of biological priors. This left a total of 181 DEGs from ACL and 373 DEGs from synovium for prior assignment.

Pathway analysis using the PANTHER classification system did not identify overrepresented pathways among DEGs identified in ligament. There were two overrepresented pathways among DEGs from case and control synovium (Table 4).

**TABLE 4:** Overrepresented pathways identified from differentially expressed genes (DEGs) derived from synovium tissue collected from ACL rupture cases and matched controls.

**PANTHER pathway**	**Number observed**	**Number expected**	**FDR *P*-value**
B lymphocyte activation	8	1.41	8.17E-03
T lymphocyte activation	9	1.64	5.91E-03

*Pathway overrepresentation was calculated using Fisher’s Exact test in PANTHER version 15.0 (Mi et al., 2013). FDR, false discovery rate.*

### Association Analysis

**FIGURE 1:**
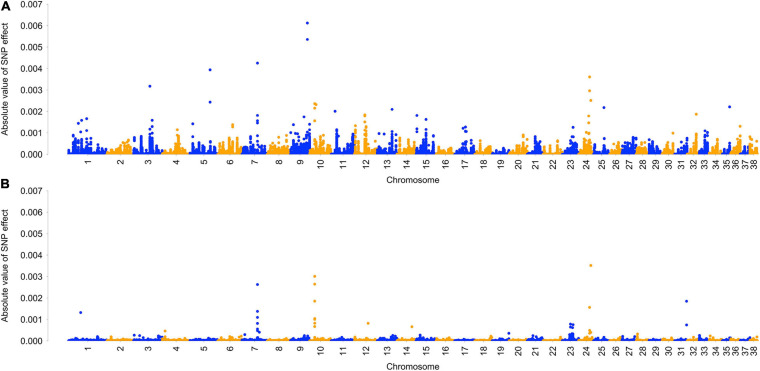
Manhattan plot of genome-wide association analysis of ACL rupture case and control dogs with and without biological priors. Plot shows average SNP effect across five reps of BayesRC algorithm **(A)** and BayesR algorithm **(B)**.

Single nucleotide polymorphism effects were averaged across five BayesRC runs. Overall, an average of 3,728 SNPs (0.8%) had some estimated effect, with the remainder of SNPs assigned to the zero-effect distribution. On average, 37 SNPs were assigned to the $0.01\,\sigma_{\text{g}}^{2}$ distribution, 361 SNPs were assigned to the $0.001\,\sigma_{\text{g}}^{2}$ distribution, and 3,330 SNPs were assigned to the $0.0001\,\sigma_{\text{g}}^{2}$ distribution. GWAS results from analysis with and without biological priors are visually represented in a Manhattan plot (Figure 1), showing five regions with largest effects on chromosomes 3, 5, 7, 9, and 24 (Supplementary Figure 2). The 50 largest SNP effects and their distance to genes are reported in Table 5. Full results are reported in Supplementary Table 4.

**TABLE 5:** The 50 largest SNP effects from Bayesian mixture model (BayesRC) association analysis that included biological priors.

**Chromosome: location**	**Class**	**SNP effect**	**Gene**	**Distance (bp)**
chr9:53865770	ACL	0.006	*FNBP1*	9,144
chr9:53871457	ACL	0.005	*FNBP1*	3,457
chr7:49455960	NA	−0.004	None	N/A
chr5:64359450	ACL	0.004	*ACSF3*	21,679
chr24:34970050	NA	0.004	None	N/A
chr3:51975977	LIT	−0.003	*ACAN*	19,131
chr24:34842049	NA	−0.003	*SULF2*	0
chr24:38868995	NA	−0.003	Non-coding transcript	467
chr5:64356666	ACL	0.002	*ACSF3*	19,554
chr10:16199198	NA	0.002	None	N/A
chr10:20536317	A&S	−0.002	*FBLN1*	0
chr35:23442178	SYN	0.002	*LRRC16A*	0
chr25:33171736	SYN	−0.002	*ADAM28*	23,934
chr10:16138674	NA	0.002	ENSCAFG00000031351	19,408
chr13:47443664	LIT	−0.002	*KDR*	0
chr11:12024840	LIT	0.002	*LOX*	0
chr32:28999310	A&S	0.002	*RPL34*	4,462
chr32:28996275	A&S	0.002	*RPL34*	0
chr12:32821658	LIT	−0.002	*COL9A1*	0
chr7:49422990	NA	−0.002	None	N/A
chr15:2674794	A&S	0.002	*ZMPSTE24*	0
chr24:32151336	LIT	0.002	*WISP2*	0
chr12:32792729	LIT	−0.002	*COL9A1*	0
chr9:43053330	ACL	−0.002	*FLOT2*	0
chr1:57856871	A&S	−0.002	*NEPN*	0
chr15:31879223	LIT	−0.002	*DCN*	0
chr3:59287831	LIT	−0.002	*SORCS2*	0
chr1:41687833	A&S	0.002	*AKAP12*	0
chr7:49415778	NA	−0.002	None	N/A
chr10:16118097	NA	0.002	None	N/A
chr12:32815853	LIT	−0.002	*COL9A1*	0
chr24:32144138	LIT	−0.002	*WISP2*	0
chr7:49426351	NA	−0.001	None	N/A
chr1:31289631	A&S	−0.001	*ABRACL*	22,185
chr5:9055237	A&S	−0.001	*PKNOX2*	0
chr9:60820860	A&S	−0.001	*DAB2IP*	0
chr9:9241561	LIT	0.001	*ITGB3*	17,351
chr6:47617815	LIT	−0.001	*COL11A1*	0
chr12:2604649	LIT	−0.001	*HLA-DPB1*	
chr36:30472199	LIT	0.001	*COL3A1*	16,392
chr3:59282629	LIT	−0.001	*SORCS2*	0
chr12:36850837	LIT	−0.001	*COL12A1*	12,221
chr6:47604275	LIT	−0.001	*COL11A1*	0
chr17:34324882	A&S	−0.001	*ARID5A*	0
chr23:34306524	NA	−0.001	*DZIP1L*	0
chr12:36852892	LIT	−0.001	*COL12A1*	14,276
chr12:36767540	LIT	−0.001	*COL12A1*	0
chr17:26672332	ACL	0.001	*FAM98A*	0
chr15:2655972	A&S	0.001	*COL9A2*	0
chr3:52046039	LIT	−0.001	*HAPLN3*	0

*SNP, single nucleotide polymorphism. Class refers to the biological prior the SNP was assigned to with ACL, differentially expressed genes in ACL; SYN, differentially expressed genes in knee synovium; A&S, differentially expressed genes in ACL and knee synovium; LIT, gene associations reported in peer-reviewed literature; NA, SNPs not assigned to biological priors. SNPs with a distance of 0 are located within the listed gene.*

### Selection Signature Analysis

**FIGURE 2:**
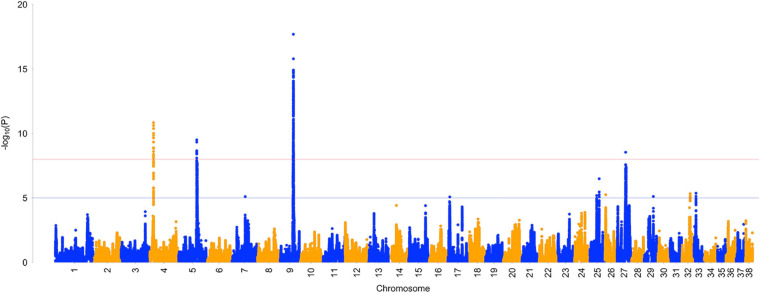
Manhattan plot of –log_10_ *P*-values from selection signature analysis using the XP-EHH test between ACL rupture case and control populations in the Labrador Retriever model. The red line denotes genome-wide significance (*P <* 1E-08). The blue line denotes suggestive significance (*P* < 1E-05).

A Manhattan plot of XP-EHH results is presented in Figure 2. Overall, 11 regions of the genome showed high levels of differentiation between case and control populations (Table 6). Significant selection signatures were identified on chromosomes 4, 5, 9, and 27. In multiple cases, haplotype boundaries overlapped associations from the GWAS analysis and/or genes that may be relevant to selection in the Labrador Retriever.

**TABLE 6:** SNPs from selection signature analysis using the XP-EHH test between ACL rupture case and control populations with *P* ≤ 1E-05.

**Chromosome: location (bp)**	**XP-EHH statistic**	**−log_10_(*P*-value)**		**Haplotype-cases (bp)**	**Haplotype-controls (bp)**	**GWAS association**	**Morphological trait**
chr9:41939206	8.755	17.689	Ancestral	38,354,858–44,845,348	38,445,025–45,354,649	No	Yes
			Derived	36,403,205–46,304,783	37,547,484–46,288,289		
chr4:12205862	−6.752	10.835	Ancestral	7,510,955–14,887,026	6,072,208–17,040,743	No	No
			Derived	6,959,664–15,047,339	6,463,425–15,725,909		
chr5:58850078	6.291	9.501	Ancestral	53,057,850–65,529,018	53,659,098–64,565,426	Yes	Yes
			Derived	54,988,840–63,453,457	54,868,687–63,041,937		
chr27:26574198	−5.938	8.541	Ancestral	23,516,127–29,439,310	23,699,703–30,620,059	No	Yes
			Derived	22,222,149–32,085,822	21,931,147–32,234,026		
chr25:34033213	5.107	6.485	Ancestral	31,661,754–35,332,812	31,715,640–35,883,264	Yes	No
			Derived	28,588,690–36,482,765	28,756,320–36,702,771		
chr33:7205917	−4.595	5.363	Ancestral	3,744,045–10,043,116	3,720,126–10,051,517	No	No
			Derived	3,883,742–9,956,115	3,828,759–9,732,898		
chr32:25969031	−4.579	5.331	Ancestral	23,509,436–27,789,317	23,844,794–28,445,312	Yes	No
			Derived	23,285,054–28,396,281	22,768,230–29,128,860		
chr26:3270682	4.539	5.248	Ancestral	1,230,631–6,838,428	488,036–8,368,487	No	Yes
			Derived	846,901–9,267,168	828,802–8,660,209		
chr29:30730571	−4.473	5.112	Ancestral	26,331,748–33,625,961	27,004,351–33,567,802	No	No
			Derived	26,328,442–32,957,785	25,878,499–33,457,544		
chr7:44727369	−4.467	5.101	Ancestral	41,121,286–47,960,527	40,077,404–49,832,418	Yes	Yes
			Derived	39,520,448–50,729,292	38,885,756–51,222,590		
chr17:8919370	4.454	5.075	Ancestral	6,224,233–11,785,115	7,985,197–11,604,799	No	No
			Derived	3,894,493–13,562,415	4,236,063–13,491,948		

*Positive XP-EHH values indicate regions that are under positive selection for the derived haplotype. Negative XP-EHH values indicate regions that are under negative selection for the derived haplotype. bp, base pairs. The ancestral haplotype is the haplotype with greater frequency than the derived haplotype. For each haplotype under selection, we report whether it overlaps with a GWAS association from this analysis and/or a gene/region that has been associated with a morphological trait in dogs that may be driving selection.*

## Discussion

Incorporation of prior biological information using the BMM algorithm BayesRC allows the user to prioritize SNPs based on biological probability of effect in GWAS analysis. This is in contrast to the more subjective decisions that are often made when evaluating GWAS results (Thompson et al., 2013). Here, we were able to identify associations within or near many relevant candidate genes for ACL rupture. Many of the largest effect SNPs were within or near genes that were either differentially expressed between ACL rupture case and control dogs or were candidate genes that have been reported previously in both human and canine studies of the genetic predisposition to ACL rupture.

To assign biological priors, we first performed RNA sequencing in ACL and synovium tissues from ACL rupture affected and matched control dogs to identify DEGs, and DEG lists were submitted for pathway enrichment analysis. While there were no overrepresented pathways identified in ACL, there were two overrepresented pathways in synovium representing genes that are expressed in B and T lymphocyte activation, clearly indicating that inflammatory response is an important differentiator between cases and controls. This is perhaps unsurprising, as ACL rupture is associated with marked lymphocytic synovitis in dogs (Little et al., 2014). It is unclear whether ACL rupture-associated synovitis is a cause or effect of ligament rupture, as signs of synovial effusion are often present on radiographs and synovitis can also be seen arthroscopically before development of complete ACL rupture and associated joint instability (Muir et al., 2011; Little et al., 2014), and these signs are predictive of future ACL rupture (Chuang et al., 2014). In humans, synovitis is associated with development and progression of OA (Hügle and Geurts, 2017). Future research warrants further investigation into the details of the genes that were significantly differentially expressed between ACL rupture cases and controls, and whether their differential expression may be related to a response unique to ACL rupture patients.

BayesRC analysis identified several regions of the genome that show association with ACL rupture in the dog model. A Manhattan plot of GWAS results shows five regions with the largest effects on chromosomes 3, 5, 7, 9, and 24. When BayesRC results are compared to a similar analysis without the inclusion of biological priors (BayesR), associations on chromosomes 7, 10 (10th largest effect in BayesRC results), and 24 remain, but we can see that many more associations are identified when biological priors are included as part of the analysis.

Intense artificial selection during breed creation may be partially responsible for the high prevalence of ACL rupture in the Labrador Retriever population. Associations that are identified in both GWAS and selection signature analysis may be more biologically significant to ACL rupture risk than regions that were identified in a single analysis. We identified 11 regions of the genome that showed evidence of selection. Some of these regions lend themselves to hypotheses over what may be driving selection in a direction that increases risk of ACL rupture. The signature on chromosome 5 is most notable. This region overlaps our GWAS association on chromosome 5, which is near the gene *ACSF3*, which was also differentially expressed in ligament. This region also overlaps the boundaries of *MC1R*, the gene responsible for black versus yellow coat color in the Labrador Retriever (Everts et al., 2000), a clear target for artificial selection. We have observed in previous work that Labrador Retrievers with yellow coat color are overrepresented among cases (Terhaar et al., 2020). *ACSF3* has also been shown to be under selection in sporting breeds (which includes the Labrador Retriever) compared to other breeds (Kim et al., 2018). In humans, *ACSF3* has been reported as a risk gene in patients with rheumatoid arthritis (RA) (Julià et al., 2017). Within the cell, mitochondria maintain a pathway for fatty acid synthesis (FAS) that is distinct from cytosolic FAS. *ACSF3* encodes the enzyme responsible for the first step of mitochondrial FAS. T-cells from patients with RA have undergone a metabolic shift to a pro-invasive, proinflammatory state that is marked by impaired glycolysis and increased cytosolic FAS (Shen et al., 2017). In our study, *ACSF3* was expressed more highly in cases, which indicates that cells in the sample were highly metabolically active (Bowman and Wolfgang, 2018). *In vitro* research suggests that increased activity of mitochondrial FAS leads to reduced glucose utilization and increased cytosolic FAS (Clay et al., 2016), which may support a proinflammatory state. Since *MC1R* and *ACSF3* are within the same haplotype in our population, selecting for coat color may select for a haplotype that affects *ACSF3* activity, or through *MC1R* itself (or both). MC1-signaling is not limited to melanin production, it also plays a role in the inflammatory system and has been shown to protect against cartilage degeneration and subchondral bone sclerosis in OA (Montero-Melendez et al., 2020). MC1 agonists are being investigated as disease-modifying treatments for a range of inflammatory diseases (Spana et al., 2019; Montero-Melendez et al., 2020). Yellow Labrador Retrievers lack functional MC1 receptors, which means that they may be predisposed to inflammation as well, and this could affect their risk of ACL rupture. Though certain people with red hair also lack functional MC1 receptors, research on the effect of MC1 signaling on the risk of inflammatory disease in people or dogs is limited. Given the findings in the present study and clear biological significance of this region, it will be important to follow up on these findings in future work.

Other signatures also illuminate hypotheses for biological mechanisms underlying ACL rupture risk. The signature on chromosome 7, which overlaps with our GWAS association, is a well-known region that contains the gene *SMAD2*, which has repeatedly been associated with weight and body size in dogs (Chase et al., 2009; Rimbault et al., 2013; Hayward et al., 2019; Plassais et al., 2019; Bannasch et al., 2020). The signature on chromosome 26 also overlaps with a region that has been associated with weight and height in dogs (Hayward et al., 2019). Multiple epidemiological studies of ACL rupture in dogs, including our own, have identified weight as a risk factor (Whitehair et al., 1993; Duval et al., 1999; Adams et al., 2011; Baker et al., 2018), though in most cases it is unclear if this is a function of obesity or body size. The signature on chromosome 9 has been identified previously in the Labrador Retriever (Wiener et al., 2017) as a region that is under selection in dogs bred for sport (hunting) versus show. Labrador Retrievers that are bred for hunting are morphologically distinct from those that are bred for show, and it is possible that these morphological differences may be contributing to risk of ACL rupture. The region on chromosome 27 overlaps the boundaries of *KRT71*, which is responsible for curled-coat in dogs (Cadieu et al., 2009). Labrador Retrievers may have a wavy coat, though this is a less desirable feature according to AKC standard.

There were several associations from GWAS analysis that did not overlap with the selection signature analysis. These associations are also important for understanding the underlying pathophysiology of ACL rupture. For example, the top association on chromosome 9 was within the gene *FNBP1*, which was also differentially expressed in ligament. *FNBP1*, which is also known as *FBP17*, encodes a protein essential for clathrin-mediated endocytosis (Kamioka et al., 2004). It is also involved in regulation of the actin filament assembly for the actin cytoskeleton, which is important for cellular migration and the maintenance of cell shape (Higgs, 2005; Aspenström, 2010). ACL fibroblasts are known to undergo cytoskeletal reorganization after a strain event to align in longitudinal orientation with the strain (Lee et al., 2005). In this study, *FNBP1* was expressed more highly in cases than controls, which may have been associated with actin reorganization in response to ACL injury. GWAS results highlighted several other genes that also have a role in actin cytoskeleton homeostasis, including *LRRC16A* (also known as *CARMIL1*) (Edwards et al., 2013), *KDR* (Luykenaar et al., 2009), *LOX* (Payne et al., 2006), *FLOT2* (Langhorst et al., 2007), *AKAP12* (Benz et al., 2019), *ABRACL* (Wang et al., 2019), *PKNOX2* (also known as *PREP2*) (Haller et al., 2004), *ITGB3* (Urbinati et al., 2012), and *SORCS2* (Deinhardt et al., 2011), which has also been reported as associated with ACL rupture in Newfoundland dogs (Baird et al., 2014b). This pattern in the association results suggests that variable actin dynamics may play a role in genetic predisposition to ACL rupture. These observations indicate there could be a heritable difference in response to injury between dogs that rupture their ACL and those that do not.

Other associations within the top 50 SNP effects point to genes involved in the extracellular matrix. Aggrecan *(ACAN)* was within the top five GWAS associations. Aggrecan plays a vital role in maintaining hydration in the extracellular matrix of collagenous tissues, including ligamentous tissue. An association between *ACAN* and ACL rupture has been reported multiple times in humans (Mannion et al., 2014; Johnson et al., 2015) and dogs (Wilke, 2010). It has also been associated with degenerative ligament disease in horses (Plaas et al., 2011). While an association between aggrecan and ACL rupture has been reported before in a GWAS from a subset of this dataset (Baker et al., 2017), we chose to keep *ACAN* in the list of candidate genes because of its connection to degenerative ligament disease across species, and because the previously reported association in the Labrador Retriever was weak (*P* = 1.07E-04). Because of this, care should be taken not to overinterpret this association. However, the strength of the association in this study as well as the body of evidence that exists to support it leads us to consider this association as additional evidence of a role for aggrecan in the pathobiology of ACL rupture. *SULF2* was also among the top five associations. *SULF2* encodes an enzyme that is important for regulation of overall balance of cartilage matrix synthesis and degradation (Otsuki et al., 2010). *SULF2* was not assigned to a biological prior, and therefore this association is derived from genetic data only. *SULF2* knockout mice develop early-onset OA in their knee joints at 6 months of age with reduced glycosaminoglycan content and lower cellularity in articular cartilage (Otsuki et al., 2008). Dogs with ACL rupture also develop early osteoarthritic changes that are typically present at the time of diagnosis (Chuang et al., 2014), and early-onset OA is common in human ACL rupture patients (Lohmander et al., 2007). An association between *SULF2* and ACL rupture has not been previously reported. Other notable extracellular matrix genes include several collagen genes *COL9A1, COL11A1, COL12A1, COL3A1*, and *COL9A2*. All of these genes were included in the candidate genes from peer-reviewed literature class, as various associations have been reported previously in human and dogs. These associations have not been previously validated in either species. *COL9A2* was also differentially expressed in both ACL and knee synovium. *COL9A2* encodes the alpha-2 chain of type IX collagen, which is crucial to the maintenance of articular cartilage. Reduced levels of type IX collagen may contribute to OA pathogenesis (Luo et al., 2017).

With the exception of the associations on chromosome 3 with *ACAN* (Wilke, 2010; Baker et al., 2017) and *SORCS2* (Baird et al., 2014a), most the associations identified in this study did not overlap with associations identified in previous GWAS for ACL rupture in dogs (Baker et al., 2017, 2018; Huang et al., 2017; Hayward et al., 2019). The use of biological priors in the present work was performed, in part, due to the inconsistencies in GWAS results across studies. We consider the current study an extension of our former work that differs in many ways. Baker et al. (2017) analyzed considerably fewer dogs (*N* = 237), used a different method for statistical analysis, and did not correct for weight or age as fixed effects, and any of these factors could explain different results. Baker et al. (2018) was a multivariate analysis aimed at answering how genetics may impact tibial morphology, and whether this has an effect on risk of ACL rupture. We expect that the effects discovered in this analysis would have smaller effects on ACL rupture as a whole compared to their effects on tibial morphology combined with ACL rupture risk (Baker et al., 2018), so it is not surprising that these results were not among the top SNP effects in the present analysis. The research that has come from Cornell University (Hayward et al., 2016, 2019; Huang et al., 2017) used a dataset that contains many dog breeds. The SNP associations identified in these studies may be reflective of ACL rupture risk that are either weaker in the Labrador Retriever, or specific to risk in other breeds. We believe that the overlap of significant GWAS results with DEGs and selection signatures speaks to the strength of the present work.

In MacLeod et al. (2016), it is noted that the Dirichlet prior in BayesRC may have greater influence on the posterior if the number of variants in one class is low relative to the rest of the dataset. The authors suggested that classes should have 1,000 variants or greater to allow the data to have strong influence on the posterior parameters, especially when there is greater uncertainty, for example, when candidate genes from reported literature are used for prior assignment. To avoid this, we made sure that >1,000 SNPs were assigned to the peer-reviewed literature class used in this study (Class LIT, Table 2). However, there were fewer than 1,000 SNPs in Class A&S, which represented genes that were differentially expressed in both ligament and synovium tissues. This was known before BayesRC analysis, and the choice was made to maintain this class for two reasons: (1) these genes were differentially expressed in both ACL and knee synovium tissues, so there is inherently less uncertainty around their candidacy and (2) because they were differentially expressed in both tissues, it seemed important to designate them separately from genes that were differentially expressed in only one tissue. Ten of the top 50 SNP effects were assigned to Class A&S, which is a relatively high number given 703/433,227 = 0.16% of SNPs were assigned to Class A&S, and these SNPs represent 20% of the top SNP effects. It is not clear whether the effect of these SNPs is due to true association with the disease, or potential bias from prior assignment, and these results should be interpreted with this in mind.

Genomic prediction is widely used in production animal populations to select individuals for breeding based on their estimated breeding value (EBV) for a desirable trait such as milk production or meat quality. Genomic prediction for complex diseases in human populations, referred to as a polygenic risk score (PRS), has received considerable attention in recent research (Chatterjee et al., 2016). Though the calculation is generally the same, the goal in human research is focused on individual risk management and personalized medicine rather than breeding decisions (Wray et al., 2019). In companion animal populations such as the dog, the EBV/PRS could be used for both breeding decisions and individual medical management. ACL rupture, in particular, is an acquired trait in dogs that may not present itself until well after a dog has given birth to or sired many litters (Witsberger et al., 2008). High-risk individuals would become candidates for a weight management program (Witsberger et al., 2008), possible delayed neutering (Torres de la Riva et al., 2013), and radiographic screening (Chuang et al., 2014). Development of a PRS for ACL rupture in the dog model is an important goal of our research (Baker et al., 2017, 2020). Incorporating biological knowledge using the BayesRC algorithm has been reported to improve accuracy of genomic prediction in livestock populations (MacLeod et al., 2016). The accuracy of genomic prediction is heavily affected by the size of the reference population, with most estimates using sample sizes well into the thousands (Van Raden et al., 2009; Liu et al., 2011; Dudbridge, 2013). We did not attempt genomic prediction as a part of this study due to the size of our reference dataset. Recruitment of additional Labrador Retrievers is underway with the intention to develop a PRS for ACL rupture in the dog model using BayesRC and/or other algorithms.

## Conclusion

Incorporation of *a priori* biological information into BMM analysis using BayesRC in a dog model of ACL rupture was able to replicate associations that were previously reported in human and dog studies, especially in collagen genes, and also identify novel genetic associations with ACL rupture. Several associations reported in human studies have been identified here, in the dog, which highlights the value of One Health medicine (Gyles, 2016), and the dog in particular as a valuable model for genomic research. The actin cytoskeleton is the basis for cellular organization and shape and is integral for a cell’s capacity to migrate. This is the first study to suggest a role for the actin cytoskeleton in risk of ACL rupture. Additionally, while *SULF2* has been implicated in onset and progression of OA, which is typically associated with ACL rupture, this is the first publication to report an association between *SULF2* and ACL rupture itself. Many of the associations we identified in this study overlap with regions of the genome that are under selection in the Labrador Retriever. These findings begin to provide an explanation for the high prevalence of ACL rupture in this breed and highlight the unintended consequences of artificial selection.

## Data Availability Statement

The RNASeq data presented in the study are deposited in the ArrayExpress repository, accession number E-MTAB-10119. The SNP data presented in this study are deposited in the European Variation Archive (EVA), project number PRJEB43243 and analysis number ERZ1743079.

## Ethics Statement

The animal study was reviewed and approved by the Animal Care Committee of the University of Wisconsin-Madison (protocols V1070 and V5463). Written informed consent was obtained from the owners for the participation of their animals in this study.

## Author Contributions

LB carried out the study and wrote the first draft of the manuscript. MM performed selection signature analysis and contributed to writing and editing of the manuscript. RM contributed to initial data analysis and editing the manuscript. MB consulted on study design, performed the RNA sequencing analysis for the experiment, and contributed to the writing and editing of the manuscript. EB and SS contributed to dog recruitment and sample collection, maintenance of the data, interpretation of the results, and reviewed the manuscript. PM designed the experiment, obtained funding for the experiment, supervised the study, and revised the manuscript. All authors have read and approved the final version of the manuscript.

## Conflict of Interest

The authors of this manuscript have the following competing interests: PM, LB and MM are named on US Patent US20160222451A1 “Method to predict heritable canine non-contact cruciate ligament rupture.” This does not alter our adherence to the journal’s policies on data sharing and materials. The remaining authors declare that the research was conducted in the absence of any commercial or financial relationships that could be construed as a potential conflict of interest.
