# Supplementary material for: Biologically Enhanced Genome-Wide Association Study Provides Further Evidence for Candidate Loci and Discovers Novel Loci That Influence Risk of Anterior Cruciate Ligament Rupture in a Dog Model
Source: Front Genet. 2021 Mar 5;12:593515. doi: 10.3389/fgene.2021.593515 (PMC7982834; doi:10.3389/fgene.2021.593515)

Supplementary Figure 1. We defined 4 biological prior classes for genome wide association analysis using BayesRC. The procedure for assigning SNPs to each class is shown. Candidate genes were defined through RNA sequencing and differential gene expression analysis of anterior cruciate ligament (ACL) and knee synovium. Three biological prior classes were defined based on the differentially expressed genes' tissue of origin ("ACL", "SYN", or "A&S"). We defined a fourth class from reported associations with anterior cruciate ligament rupture and tendinopathy in peer-reviewed literature ("LIT"). SNPs that were not within or near candidate genes were assigned to a separate class ("NA").

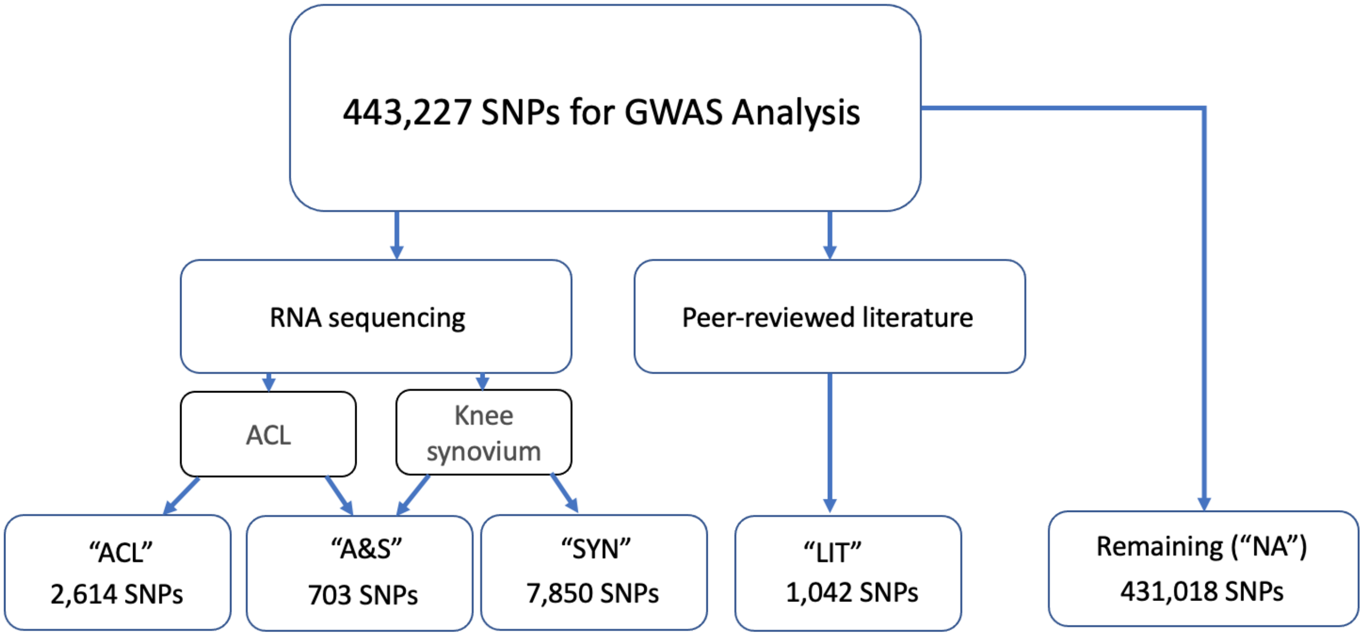

Supplement: Supplementary file 1 [file Image_1.pdf]
