# Supplementary material for: Biologically Enhanced Genome-Wide Association Study Provides Further Evidence for Candidate Loci and Discovers Novel Loci That Influence Risk of Anterior Cruciate Ligament Rupture in a Dog Model
Source: Front Genet. 2021 Mar 5;12:593515. doi: 10.3389/fgene.2021.593515 (PMC7982834; doi:10.3389/fgene.2021.593515)
Supplement: Supplementary file 2 [file Image_2.pdf]

Supplementary Figure 2. Top 5 GWAS associations from BayesRC analysis with linkage disequilibrium and gene annotation information. Plots were generated using LocusZoom standalone software v1.4 (https://genome.sph.umich.edu/wiki/LocusZoom_Standalone) and a custom SQLite database created using canFam 3.1 NCBI RefSeq gene annotations downloaded from the UCSC Table Browser (https://genome.ucsc.edu/cgi-bin/hgTables).

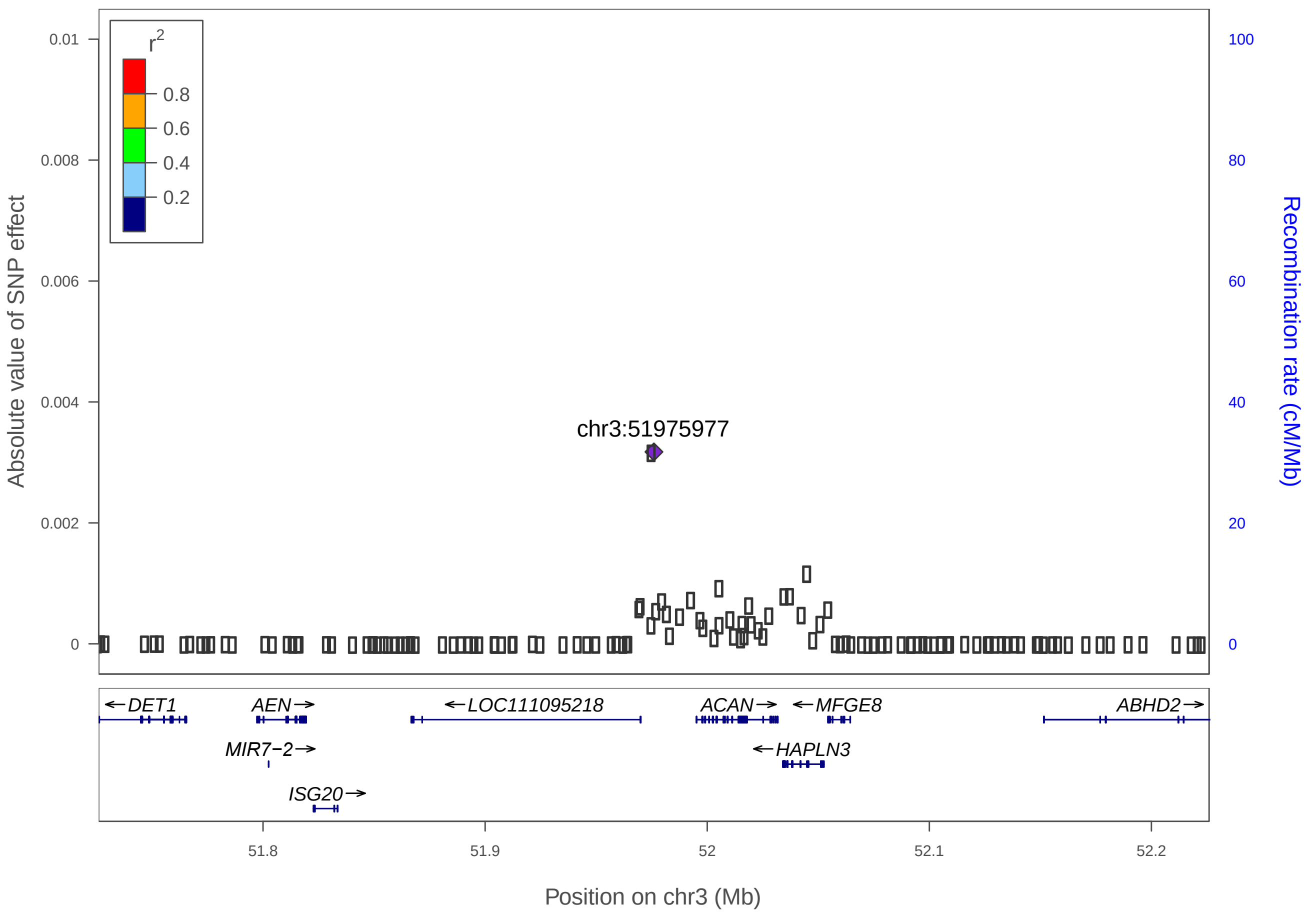

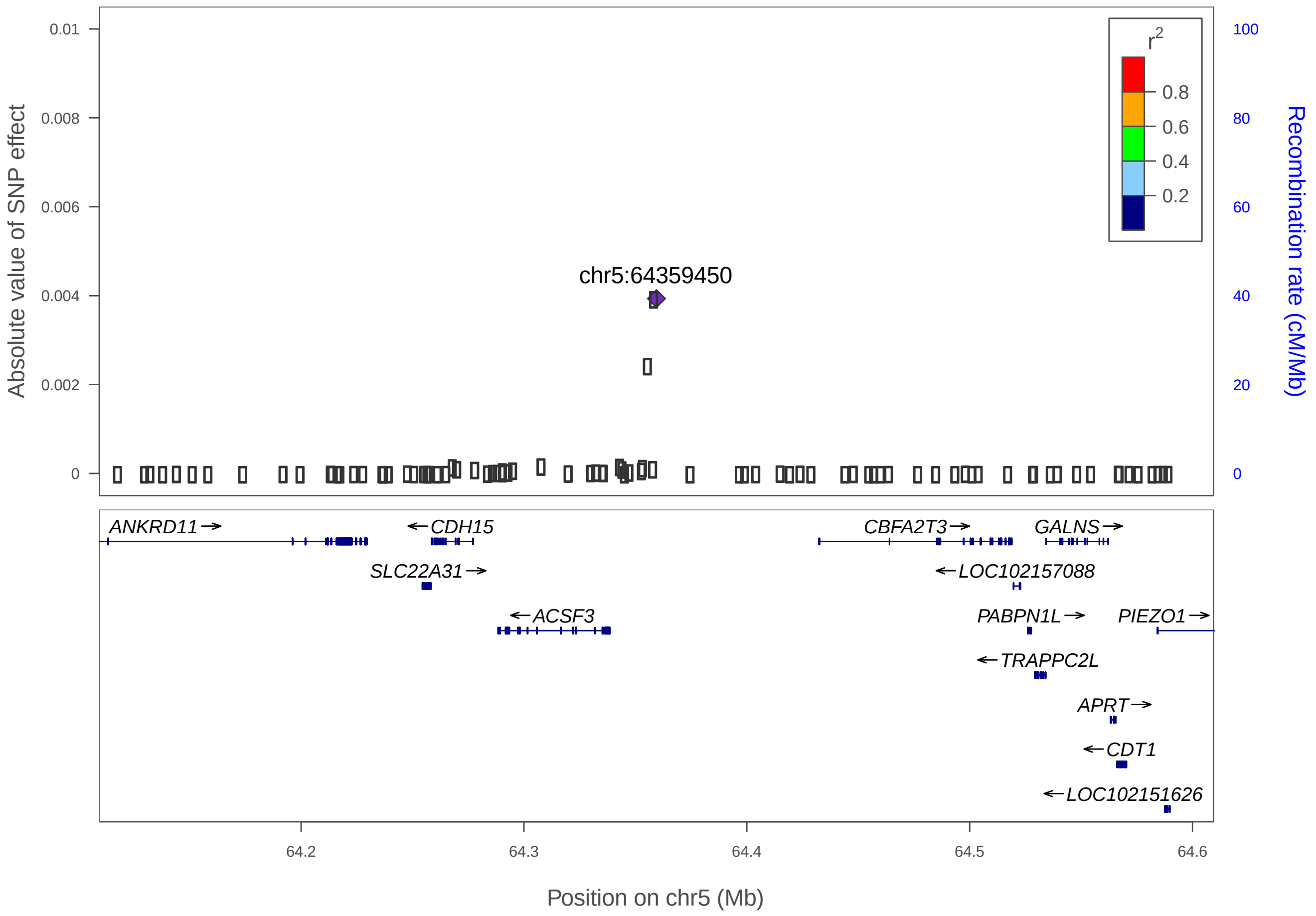

Absolute value of SNP effect
0.01
0.008
0.006
0.004
0.002
0
chr5:64359450
r2
0.8
0.6
0.4
0.2
100
80
60
40
20
0
Recombination rate (cM/Mb)
ANKRD11
CDH15
SLC22A31
ACSF3
CBFA2T3
GALNS
LOC102157088
PABPN1L
PIEZO1
TRAPPC2L
APRT
CDT1
LOC102151626
64.2
64.3
64.4
64.5
64.6
Position on chr5 (Mb)

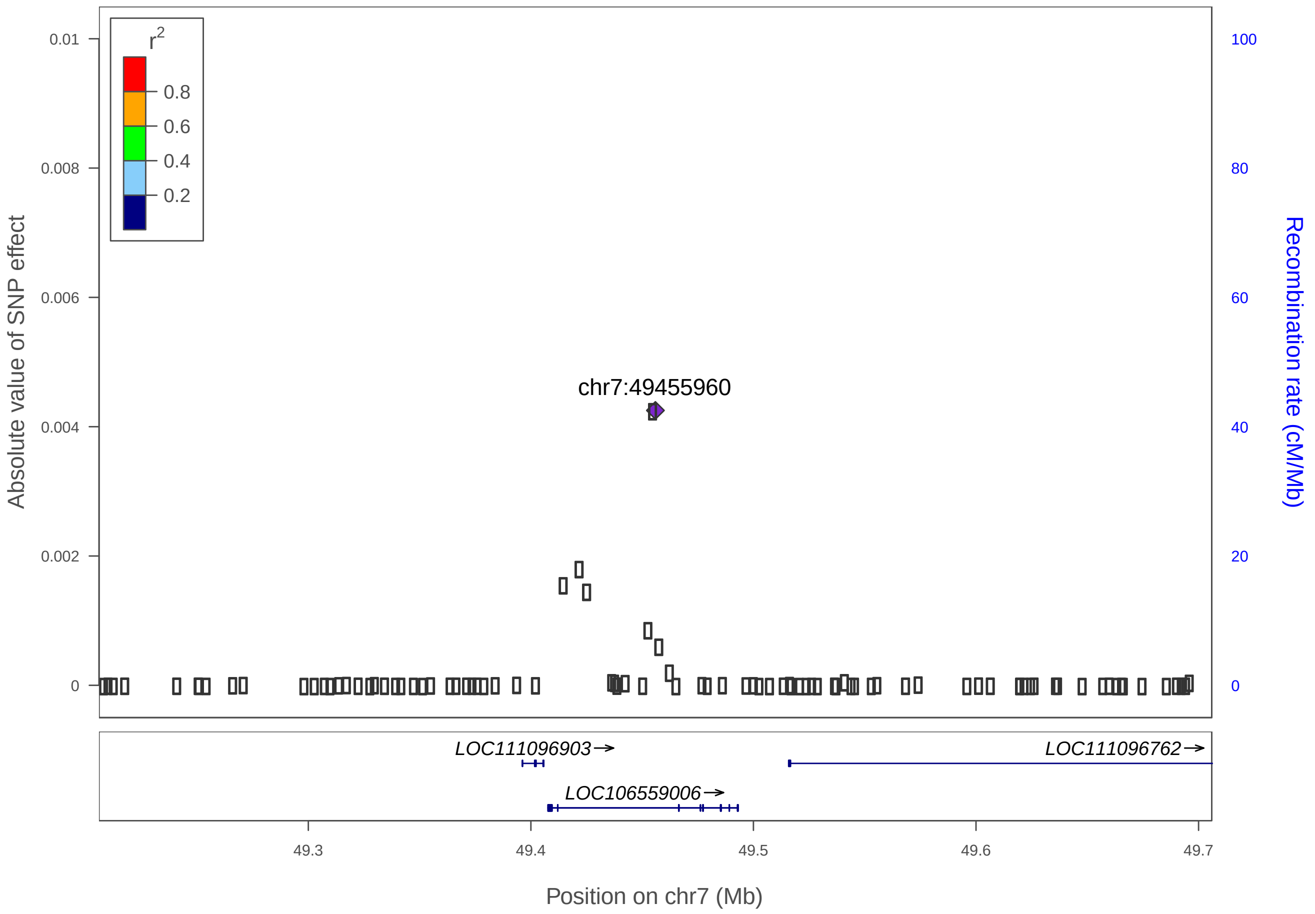

r2
0.8
0.6
0.4
0.2
0.01
0.008
0.006
0.004
0.002
0
Absolute value of SNP effect
100
80
60
40
20
0
Recombination rate (cM/Mb)
chr7:49455960
LOC111096903
LOC111096762
LOC106559006
49.3
49.4
49.5
49.6
49.7
Position on chr7 (Mb)

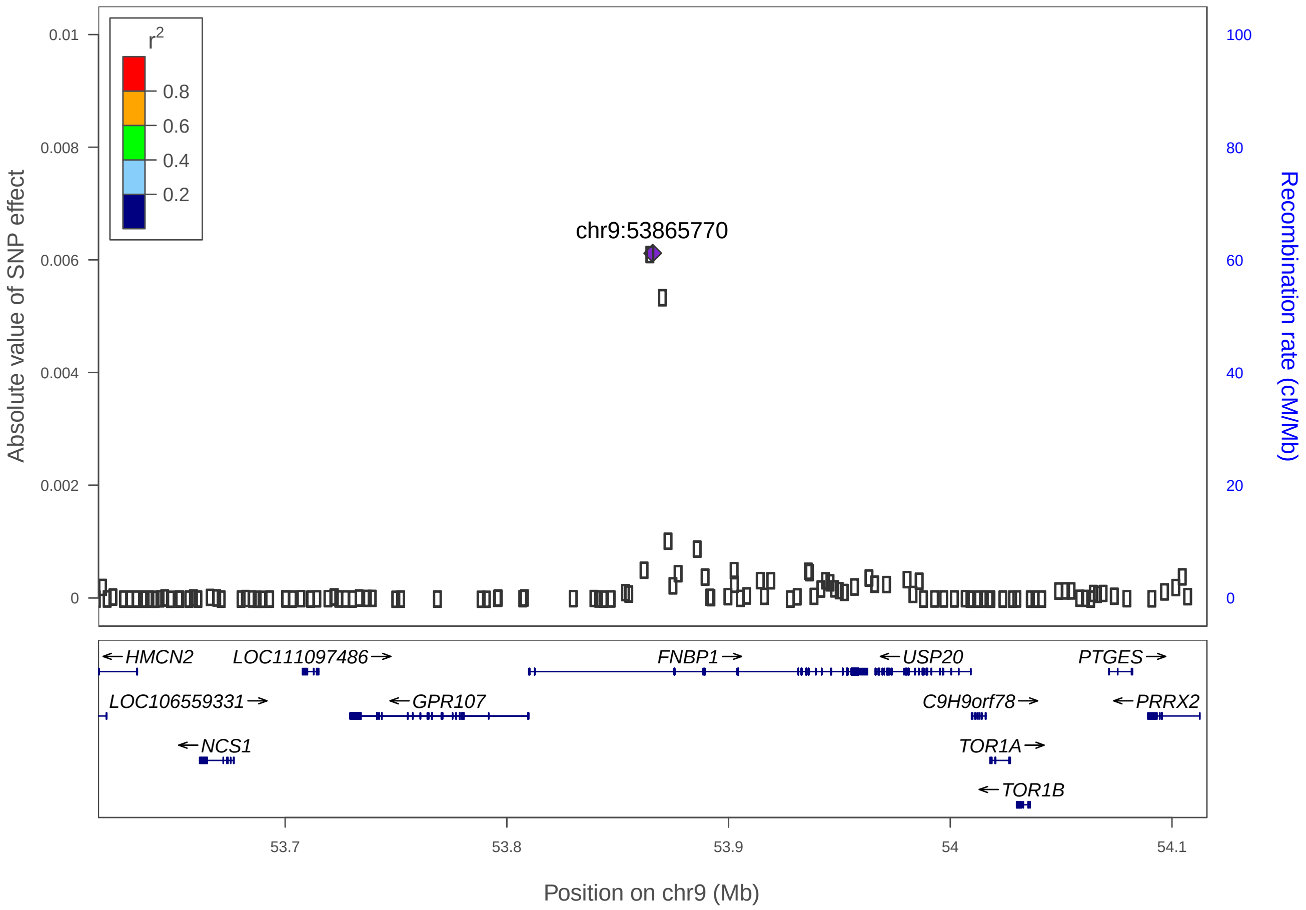

r2
0.8
0.6
0.4
0.2
chr9:53865770
Absolute value of SNP effect
0.01
0.008
0.006
0.004
0.002
0
Recombination rate (cM/Mb)
100
80
60
40
20
0
HMCN2
LOC111097486
FNBP1
USP20
PTGES
LOC106559331
GPR107
C9H9orf78
PRRX2
NCS1
TOR1A
TOR1B
53.7
53.8
53.9
54
54.1
Position on chr9 (Mb)

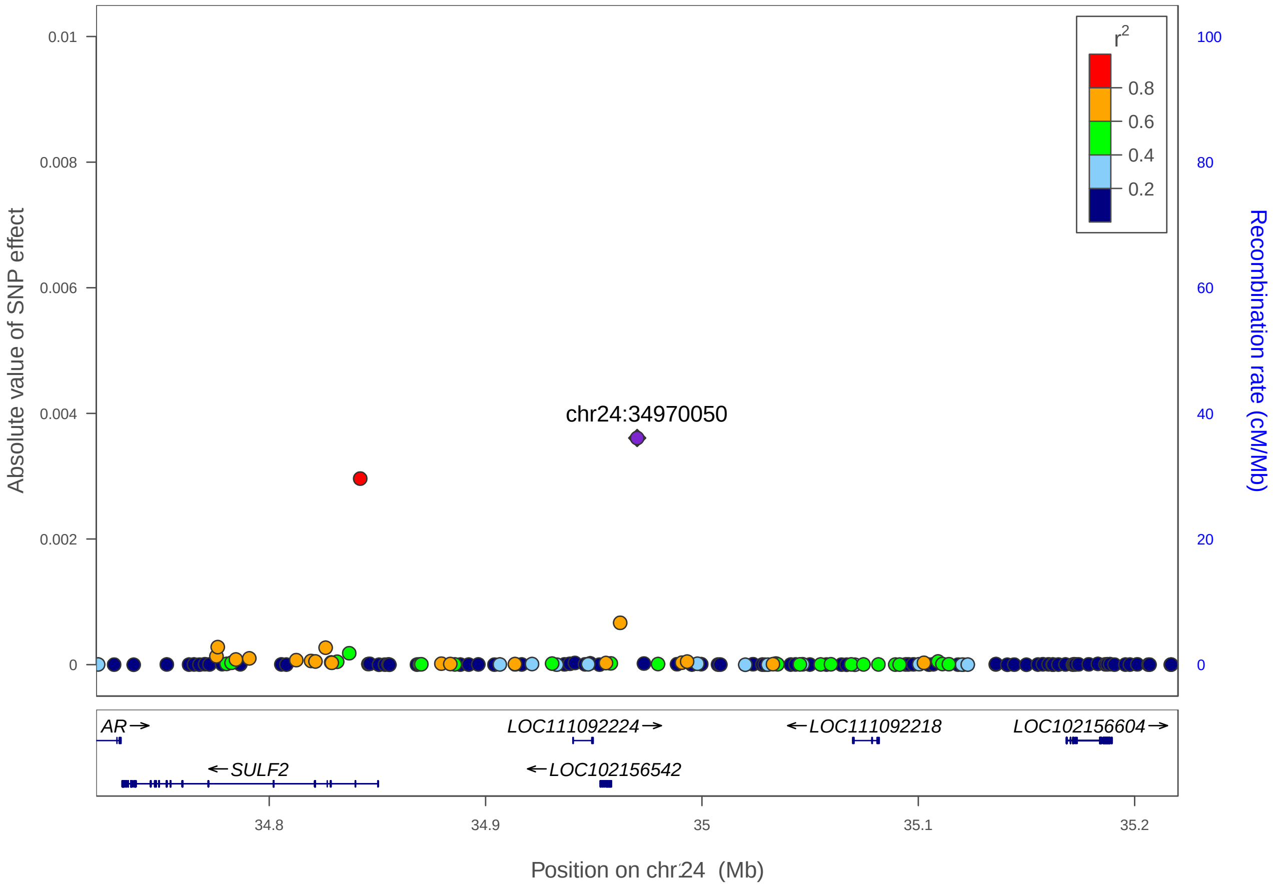

Absolute value of SNP effect
Recombination rate (cM/Mb)
r2
0.8
0.6
0.4
0.2
0.01
0.008
0.006
0.004
0.002
0
100
80
60
40
20
0
chr24:34970050
AR
SULF2
LOC111092224
LOC102156542
LOC111092218
LOC102156604
34.8
34.9
35
35.1
35.2
Position on chr24 (Mb)
