# Supplementary material for: Biologically Enhanced Genome-Wide Association Study Provides Further Evidence for Candidate Loci and Discovers Novel Loci That Influence Risk of Anterior Cruciate Ligament Rupture in a Dog Model
Source: Front Genet. 2021 Mar 5;12:593515. doi: 10.3389/fgene.2021.593515 (PMC7982834; doi:10.3389/fgene.2021.593515)
Supplement: Supplementary file 3 [file Table_1.DOCX]

**S1 Table. Accuracy of imputation of Labrador Retriever SNPs based on comparison to whole-genome sequencing data**

| **Chromosome** | **Number of SNPs compared** | **Imputation Accuracy (%)** |
| --- | --- | --- |
| 1 | 39255 | 97.6 |
| 2 | 26006 | 97.7 |
| 3 | 31771 | 97.4 |
| 4 | 30628 | 97.6 |
| 5 | 29856 | 97.6 |
| 6 | 24688 | 97.3 |
| 7 | 27319 | 97.8 |
| 8 | 24316 | 97.0 |
| 9 | 18341 | 97.0 |
| 10 | 21985 | 97.3 |
| 11 | 23202 | 97.5 |
| 12 | 25984 | 97.6 |
| 13 | 22465 | 97.8 |
| 14 | 20611 | 97.4 |
| 15 | 20975 | 97.4 |
| 16 | 19422 | 97.3 |
| 17 | 21960 | 97.6 |
| 18 | 17797 | 97.0 |
| 19 | 18960 | 97.4 |
| 20 | 18045 | 97.3 |
| 21 | 16702 | 97.1 |
| 22 | 22044 | 97.4 |
| 23 | 18252 | 97.7 |
| 24 | 16110 | 97.4 |
| 25 | 17367 | 97.3 |
| 26 | 12405 | 96.4 |
| 27 | 15958 | 97.4 |
| 28 | 14206 | 97.3 |
| 29 | 14974 | 97.3 |
| 30 | 14199 | 97.1 |
| 31 | 8935 | 94.4 |
| 32 | 9000 | 91.2 |
| 33 | 11564 | 97.0 |
| 34 | 14642 | 97.5 |
| 35 | 10504 | 97.7 |
| 36 | 11751 | 97.2 |
| 37 | 11079 | 97.2 |
| 38 | 9744 | 96.2 |
| X | 14864 | 85.5 |
