# Supplementary material for: Biologically Enhanced Genome-Wide Association Study Provides Further Evidence for Candidate Loci and Discovers Novel Loci That Influence Risk of Anterior Cruciate Ligament Rupture in a Dog Model
Source: Front Genet. 2021 Mar 5;12:593515. doi: 10.3389/fgene.2021.593515 (PMC7982834; doi:10.3389/fgene.2021.593515)
Supplement: Supplementary file 4 [file Table_2.DOCX]

**Supplementary Table S2**. Differentially expressed genes identified through RNA sequencing of anterior cruciate ligament tissue from dogs that were affected and unaffected with anterior cruciate ligament rupture.

| **Ensembl ID** | **Gene Name** | **LogFC** | ***P*** | **Adjusted *P*** |
| --- | --- | --- | --- | --- |
| ENSCAFG00000000804 | *FBLN1* | -4.865298521 | 1.90E-07 | 2.60E-03 |
| ENSCAFG00000002533 | *SEC61B* | 1.322113110 | 6.27E-07 | 4.30E-03 |
| ENSCAFG00000013476 | *PDK3* | 1.128768499 | 9.63E-06 | 2.13E-02 |
| ENSCAFG00000000419 | *AKAP12* | -3.44879101 | 9.71E-06 | 2.13E-02 |
| ENSCAFG00000005334 | *GPR180* | 1.549014410 | 1.62E-05 | 2.13E-02 |
| ENSCAFG00000008716 |  | 2.221296240 | 1.63E-05 | 2.13E-02 |
| ENSCAFG00000000418 | *MDM2* | 1.134421201 | 1.74E-05 | 2.13E-02 |
| ENSCAFG00000000931 | *NUS1* | 1.028586889 | 1.80E-05 | 2.13E-02 |
| ENSCAFG00000003945 | *DENND2A* | -1.763494852 | 1.84E-05 | 2.13E-02 |
| ENSCAFG00000004017 | *ZNF438* | -1.367547562 | 1.86E-05 | 2.13E-02 |
| ENSCAFG00000010860 | *ERGIC2* | 0.839007356 | 2.01E-05 | 2.13E-02 |
| ENSCAFG00000018117 | *CHRDL1* | -3.708275754 | 2.01E-05 | 2.13E-02 |
| ENSCAFG00000000352 | *XPOT* | 1.038498199 | 2.02E-05 | 2.13E-02 |
| ENSCAFG00000001402 | *KDELR3* | 1.964656167 | 2.98E-05 | 2.62E-02 |
| ENSCAFG00000005283 | *CLIP4* | -2.619954713 | 3.05E-05 | 2.62E-02 |
| ENSCAFG00000014713 | *REX1BD* | 2.462479119 | 3.06E-05 | 2.62E-02 |
| ENSCAFG00000008975 | *THBS4* | -3.156416687 | 3.62E-05 | 2.67E-02 |
| ENSCAFG00000013416 | *MTX2* | 0.979339824 | 3.79E-05 | 2.67E-02 |
| ENSCAFG00000003663 | *GSN* | -2.72132796 | 3.88E-05 | 2.67E-02 |
| ENSCAFG00000000868 | *SMOC2* | 3.684567283 | 3.89E-05 | 2.67E-02 |
| ENSCAFG00000031126 | *HSPA13* | 0.983470216 | 4.12E-05 | 2.69E-02 |
| ENSCAFG00000010549 | *GLRX2* | 1.143524606 | 4.59E-05 | 2.79E-02 |
| ENSCAFG00000008713 | *NUCB2* | 1.183109508 | 4.69E-05 | 2.79E-02 |
| ENSCAFG00000017626 | *FAXDC2* | -1.046463422 | 5.34E-05 | 2.91E-02 |
| ENSCAFG00000009210 | *R3HCC1* | 2.644837866 | 5.47E-05 | 2.91E-02 |
| ENSCAFG00000008467 | *EDIL3* | 3.684285905 | 5.52E-05 | 2.91E-02 |
| ENSCAFG00000017318 | *EBF1* | -1.216185198 | 5.73E-05 | 2.91E-02 |
| ENSCAFG00000005738 | *SLC30A6* | 1.023971250 | 6.38E-05 | 2.98E-02 |
| ENSCAFG00000010871 | *LRP5* | -1.516455192 | 6.46E-05 | 2.98E-02 |
| ENSCAFG00000012072 | *SEC22A* | 1.039821053 | 6.68E-05 | 2.98E-02 |
| ENSCAFG00000014698 | *CRLF1* | 2.617791261 | 7.00E-05 | 2.98E-02 |
| ENSCAFG00000008236 |  | 2.580785888 | 7.16E-05 | 2.98E-02 |
| ENSCAFG00000006124 | *ALG5* | 0.764205656 | 7.18E-05 | 2.98E-02 |
| ENSCAFG00000004456 | *NDUFB6* | 0.818638387 | 7.56E-05 | 3.00E-02 |
| ENSCAFG00000003679 | *C15H16orf87* | 1.111308853 | 8.24E-05 | 3.00E-02 |
| ENSCAFG00000005875 | *FAM98A* | 0.885355314 | 8.61E-05 | 3.00E-02 |
| ENSCAFG00000011286 | *OSTC* | 1.005118927 | 9.73E-05 | 3.00E-02 |
| ENSCAFG00000001246 | *PTCH1* | -1.520847408 | 1.00E-04 | 3.00E-02 |
| ENSCAFG00000030025 | *FGL2* | -2.175822637 | 1.02E-04 | 3.00E-02 |
| ENSCAFG00000009847 | *PCIF1* | -0.857346142 | 1.03E-04 | 3.00E-02 |
| ENSCAFG00000014799 |  | -1.555158201 | 1.05E-04 | 3.00E-02 |
| ENSCAFG00000009254 | *GALNT5* | 1.670353869 | 1.07E-04 | 3.00E-02 |
| ENSCAFG00000010582 | *BOC* | -1.629248107 | 1.07E-04 | 3.00E-02 |
| ENSCAFG00000007424 | *SWAP70* | -1.137616406 | 1.08E-04 | 3.00E-02 |
| ENSCAFG00000001550 | *FOXRED2* | -2.399843154 | 1.09E-04 | 3.00E-02 |
| ENSCAFG00000007260 | *MTFR1* | 1.072563043 | 1.10E-04 | 3.00E-02 |
| ENSCAFG00000028869 | *GPX8* | 1.521969379 | 1.12E-04 | 3.00E-02 |
| ENSCAFG00000009652 | *TWF1* | 0.877094457 | 1.13E-04 | 3.00E-02 |
| ENSCAFG00000013708 | *KANSL1* | -0.711070306 | 1.14E-04 | 3.00E-02 |
| ENSCAFG00000012256 | *GOLT1B* | 1.230423645 | 1.14E-04 | 3.00E-02 |
| ENSCAFG00000000163 | *STAT6* | -0.833992182 | 1.17E-04 | 3.00E-02 |
| ENSCAFG00000014687 | *TMEM59L* | 3.065963559 | 1.17E-04 | 3.00E-02 |
| ENSCAFG00000032515 | *ARPP19* | 0.840004442 | 1.18E-04 | 3.00E-02 |
| ENSCAFG00000006546 | *ARID5A* | -1.434106604 | 1.20E-04 | 3.00E-02 |
| ENSCAFG00000009732 | *TSPAN2* | 2.460603086 | 1.21E-04 | 3.00E-02 |
| ENSCAFG00000002392 | *ZSCAN18* | -1.720740163 | 1.24E-04 | 3.03E-02 |
| ENSCAFG00000029673 |  | 0.818162127 | 1.28E-04 | 3.06E-02 |
| ENSCAFG00000002816 | *MPP6* | 2.873557958 | 1.30E-04 | 3.06E-02 |
| ENSCAFG00000023142 | *INSYN2B* | -5.937195434 | 1.33E-04 | 3.06E-02 |
| ENSCAFG00000016848 |  | -1.871508093 | 1.35E-04 | 3.06E-02 |
| ENSCAFG00000017740 | *TBX4* | -2.734153411 | 1.36E-04 | 3.06E-02 |
| ENSCAFG00000010307 | *EIF4E* | 0.758733732 | 1.44E-04 | 3.18E-02 |
| ENSCAFG00000010924 | *PKNOX2* | -2.035401793 | 1.52E-04 | 3.31E-02 |
| ENSCAFG00000011666 | *HSPA8* | 1.427846873 | 1.57E-04 | 3.34E-02 |
| ENSCAFG00000010696 | *ADD3* | -1.387568508 | 1.59E-04 | 3.34E-02 |
| ENSCAFG00000009568 | *CLIC6* | 4.027412221 | 1.65E-04 | 3.34E-02 |
| ENSCAFG00000007392 | *CENPK* | 3.450862099 | 1.66E-04 | 3.34E-02 |
| ENSCAFG00000030429 | *PDCD2* | 0.815213510 | 1.69E-04 | 3.34E-02 |
| ENSCAFG00000001798 | *SLC30A9* | 0.712676126 | 1.73E-04 | 3.34E-02 |
| ENSCAFG00000008101 | *PLOD2* | 2.353752870 | 1.74E-04 | 3.34E-02 |
| ENSCAFG00000010821 | *TMTC1* | -2.114669129 | 1.75E-04 | 3.34E-02 |
| ENSCAFG00000011405 | *TXNIP* | -1.702062816 | 1.75E-04 | 3.34E-02 |
| ENSCAFG00000007009 | *TMEM68* | 1.000476571 | 1.80E-04 | 3.37E-02 |
| ENSCAFG00000005677 | *ARAP1* | -1.163438177 | 1.91E-04 | 3.51E-02 |
| ENSCAFG00000000008 | *TXNL4A* | 0.917872917 | 1.92E-04 | 3.51E-02 |
| ENSCAFG00000003683 | *ORC6* | 2.227295941 | 2.00E-04 | 3.55E-02 |
| ENSCAFG00000014750 | *ERO1A* | 1.027776103 | 2.00E-04 | 3.55E-02 |
| ENSCAFG00000023017 | *SLC35E3* | 1.199408460 | 2.04E-04 | 3.55E-02 |
| ENSCAFG00000028843 | *MAP1LC3C* | 4.798976230 | 2.05E-04 | 3.55E-02 |
| ENSCAFG00000011659 | *ATP9A* | -2.44242906 | 2.10E-04 | 3.55E-02 |
| ENSCAFG00000007153 | *RAB2A* | 0.709283281 | 2.11E-04 | 3.55E-02 |
| ENSCAFG00000013087 | *PATZ1* | -0.967171053 | 2.14E-04 | 3.55E-02 |
| ENSCAFG00000016793 | *ZNF646* | -1.059361913 | 2.15E-04 | 3.55E-02 |
| ENSCAFG00000007731 | *ATP1B3* | 1.165518109 | 2.20E-04 | 3.59E-02 |
| ENSCAFG00000004823 |  | 0.857286938 | 2.25E-04 | 3.60E-02 |
| ENSCAFG00000008224 | *ANKRA2* | 0.814271570 | 2.28E-04 | 3.60E-02 |
| ENSCAFG00000007666 | *EIF4G2* | 1.030988976 | 2.29E-04 | 3.60E-02 |
| ENSCAFG00000013774 | *MAP3K14* | -1.463889136 | 2.35E-04 | 3.63E-02 |
| ENSCAFG00000015978 | *UGDH* | 1.780835299 | 2.37E-04 | 3.63E-02 |
| ENSCAFG00000030867 | *HPCAL4* | 2.072374947 | 2.41E-04 | 3.63E-02 |
| ENSCAFG00000000105 | *MALT1* | -0.971445358 | 2.45E-04 | 3.63E-02 |
| ENSCAFG00000007878 | *SLC43A3* | -1.032525583 | 2.46E-04 | 3.63E-02 |
| ENSCAFG00000005637 |  | 0.645946107 | 2.49E-04 | 3.63E-02 |
| ENSCAFG00000029011 | *CD164* | 0.813321513 | 2.51E-04 | 3.63E-02 |
| ENSCAFG00000002774 | *ZNF462* | -1.50267696 | 2.52E-04 | 3.63E-02 |
| ENSCAFG00000002999 | *ZMPSTE24* | 0.732205956 | 2.60E-04 | 3.71E-02 |
| ENSCAFG00000031469 |  | -1.791450258 | 2.63E-04 | 3.72E-02 |
| ENSCAFG00000017665 | *VMP1* | 1.339660928 | 2.68E-04 | 3.73E-02 |
| ENSCAFG00000011859 | *SPC25* | 1.464729622 | 2.70E-04 | 3.73E-02 |
| ENSCAFG00000006638 | *SNAI2* | 1.308879576 | 2.85E-04 | 3.91E-02 |
| ENSCAFG00000028508 | *CD59* | 1.643212524 | 2.96E-04 | 3.95E-02 |
| ENSCAFG00000018638 | *KSR1* | -1.508558569 | 2.97E-04 | 3.95E-02 |
| ENSCAFG00000028589 |  | -1.858620544 | 2.97E-04 | 3.95E-02 |
| ENSCAFG00000001800 | *TMEM263* | 1.232378725 | 3.01E-04 | 3.96E-02 |
| ENSCAFG00000003905 | *AMD1* | 0.858416427 | 3.11E-04 | 3.96E-02 |
| ENSCAFG00000018879 |  | 0.958044776 | 3.14E-04 | 3.96E-02 |
| ENSCAFG00000006175 | *CDC25B* | -1.785199129 | 3.14E-04 | 3.96E-02 |
| ENSCAFG00000004204 | *PSMC2* | 0.621414864 | 3.15E-04 | 3.96E-02 |
| ENSCAFG00000024922 | *ATP5PF* | 0.793536737 | 3.15E-04 | 3.96E-02 |
| ENSCAFG00000015815 | *ADSS* | 1.031765794 | 3.20E-04 | 3.98E-02 |
| ENSCAFG00000017798 | *MORF4L2* | 0.931875811 | 3.25E-04 | 3.98E-02 |
| ENSCAFG00000019134 | *PPL* | -3.313895119 | 3.26E-04 | 3.98E-02 |
| ENSCAFG00000037738 |  | -1.96757667 | 3.36E-04 | 4.00E-02 |
| ENSCAFG00000018842 | *PHF12* | -0.6946492 | 3.36E-04 | 4.00E-02 |
| ENSCAFG00000028827 | *UBE2V2* | 0.790754345 | 3.40E-04 | 4.00E-02 |
| ENSCAFG00000019901 | *SLC7A5* | 2.323713647 | 3.40E-04 | 4.00E-02 |
| ENSCAFG00000014222 | *IFT80* | 0.797618495 | 3.41E-04 | 4.00E-02 |
| ENSCAFG00000017792 |  | 0.932383622 | 3.49E-04 | 4.06E-02 |
| ENSCAFG00000012605 | *ZNF687* | -1.016218362 | 3.56E-04 | 4.10E-02 |
| ENSCAFG00000000677 | *LRP12* | 1.188723435 | 3.63E-04 | 4.14E-02 |
| ENSCAFG00000013826 | *SEC23A* | 1.372167964 | 3.66E-04 | 4.15E-02 |
| ENSCAFG00000001507 | *NRF1* | -0.87001426 | 3.77E-04 | 4.19E-02 |
| ENSCAFG00000008487 | *PDE3B* | -2.384856882 | 3.85E-04 | 4.19E-02 |
| ENSCAFG00000017556 | *TEX14* | -1.528913712 | 3.86E-04 | 4.19E-02 |
| ENSCAFG00000009187 | *LOXL2* | 3.316328965 | 3.88E-04 | 4.19E-02 |
| ENSCAFG00000008738 | *TM9SF3* | 0.641813657 | 3.93E-04 | 4.19E-02 |
| ENSCAFG00000007266 | *HECTD2* | 1.176405837 | 3.94E-04 | 4.19E-02 |
| ENSCAFG00000007074 | *IMPAD1* | 1.119269053 | 3.95E-04 | 4.19E-02 |
| ENSCAFG00000003476 | *BLVRA* | 1.088794138 | 3.97E-04 | 4.19E-02 |
| ENSCAFG00000012672 | *SCFD1* | 0.771797602 | 3.99E-04 | 4.19E-02 |
| ENSCAFG00000005520 | *SNRPB2* | 0.628310836 | 4.02E-04 | 4.19E-02 |
| ENSCAFG00000007761 | *IRF2* | -0.76941207 | 4.04E-04 | 4.19E-02 |
| ENSCAFG00000013773 | *BCR* | -0.888297127 | 4.10E-04 | 4.22E-02 |
| ENSCAFG00000012449 | *USP53* | -1.626748582 | 4.19E-04 | 4.22E-02 |
| ENSCAFG00000017515 | *NRDE2* | -0.705657071 | 4.23E-04 | 4.22E-02 |
| ENSCAFG00000003198 | *FKBP15* | -0.741145301 | 4.24E-04 | 4.22E-02 |
| ENSCAFG00000005820 | *ACSS3* | -1.318294285 | 4.25E-04 | 4.22E-02 |
| ENSCAFG00000018147 | *SS18* | 0.757596900 | 4.29E-04 | 4.22E-02 |
| ENSCAFG00000018588 | *L3MBTL4* | -2.360012768 | 4.30E-04 | 4.22E-02 |
| ENSCAFG00000019849 | *ACSF3* | -1.196254746 | 4.34E-04 | 4.22E-02 |
| ENSCAFG00000029086 | *ARF4* | 0.855031976 | 4.34E-04 | 4.22E-02 |
| ENSCAFG00000011167 | *PAPSS1* | 1.283284707 | 4.44E-04 | 4.24E-02 |
| ENSCAFG00000015564 | *JKAMP* | 0.869160357 | 4.46E-04 | 4.24E-02 |
| ENSCAFG00000006917 | *MAP3K1* | -1.067656349 | 4.47E-04 | 4.24E-02 |
| ENSCAFG00000010877 | *ABCA9* | -3.769425108 | 4.52E-04 | 4.24E-02 |
| ENSCAFG00000029752 | *C11H5orf15* | 1.024215326 | 4.53E-04 | 4.24E-02 |
| ENSCAFG00000002856 | *CYCS* | 1.163527903 | 4.60E-04 | 4.24E-02 |
| ENSCAFG00000009978 | *ETS2* | -1.408580212 | 4.61E-04 | 4.24E-02 |
| ENSCAFG00000017192 | *HMMR* | 1.180278706 | 4.61E-04 | 4.24E-02 |
| ENSCAFG00000006988 | *MRPL15* | 0.629832258 | 4.70E-04 | 4.25E-02 |
| ENSCAFG00000015346 | *STAT5A* | -0.558222386 | 4.71E-04 | 4.25E-02 |
| ENSCAFG00000009031 | *CMYA5* | -1.733577253 | 4.72E-04 | 4.25E-02 |
| ENSCAFG00000016173 | *THRA* | -1.066742116 | 4.78E-04 | 4.26E-02 |
| ENSCAFG00000011099 | *AMZ2* | 1.185912741 | 4.79E-04 | 4.26E-02 |
| ENSCAFG00000031859 | *FKBP7* | 1.577556967 | 4.96E-04 | 4.36E-02 |
| ENSCAFG00000019645 | *VAMP7* | 0.680766688 | 4.98E-04 | 4.36E-02 |
| ENSCAFG00000016452 |  | 0.885333496 | 5.00E-04 | 4.36E-02 |
| ENSCAFG00000019762 | *SURF4* | 0.980846795 | 5.04E-04 | 4.36E-02 |
| ENSCAFG00000018617 | *RAVER2* | -1.693768558 | 5.06E-04 | 4.36E-02 |
| ENSCAFG00000010550 | *ZBTB8OS* | 0.699167816 | 5.12E-04 | 4.38E-02 |
| ENSCAFG00000011598 |  | 1.365035428 | 5.23E-04 | 4.45E-02 |
| ENSCAFG00000012909 | *ZNF592* | -1.036116116 | 5.34E-04 | 4.50E-02 |
| ENSCAFG00000000281 | *ABRACL* | 0.985115102 | 5.40E-04 | 4.50E-02 |
| ENSCAFG00000017296 | *RNF145* | 1.048101251 | 5.40E-04 | 4.50E-02 |
| ENSCAFG00000023022 |  | -2.421793943 | 5.45E-04 | 4.50E-02 |
| ENSCAFG00000014358 | *CASK* | 1.578716228 | 5.45E-04 | 4.50E-02 |
| ENSCAFG00000035640 |  | -3.113588697 | 5.54E-04 | 4.54E-02 |
| ENSCAFG00000002994 | *NT5E* | 1.329662017 | 5.68E-04 | 4.60E-02 |
| ENSCAFG00000016168 | *BID* | 0.795879107 | 5.68E-04 | 4.60E-02 |
| ENSCAFG00000031507 |  | -3.18148177 | 5.83E-04 | 4.69E-02 |
| ENSCAFG00000006680 | *LRIG1* | -1.465260818 | 5.87E-04 | 4.69E-02 |
| ENSCAFG00000019738 | *ATP5PB* | 0.649997250 | 5.91E-04 | 4.69E-02 |
| ENSCAFG00000014600 | *BRCA1* | 1.880013822 | 5.96E-04 | 4.69E-02 |
| ENSCAFG00000005732 | *BAHCC1* | -1.3261622 | 5.96E-04 | 4.69E-02 |
| ENSCAFG00000017616 | *LARP6* | -1.606357343 | 6.03E-04 | 4.72E-02 |
| ENSCAFG00000009232 | *AMIGO2* | -4.821536265 | 6.20E-04 | 4.81E-02 |
| ENSCAFG00000007200 | *SLC20A1* | 1.368397610 | 6.21E-04 | 4.81E-02 |
| ENSCAFG00000019827 | *AMIGO1* | -2.082186402 | 6.39E-04 | 4.86E-02 |
| ENSCAFG00000015300 | *SPPL2A* | 0.799344451 | 6.40E-04 | 4.86E-02 |
| ENSCAFG00000019963 | *FNBP1* | -0.852334276 | 6.57E-04 | 4.86E-02 |
| ENSCAFG00000019251 | *CREBBP* | -0.747136444 | 6.59E-04 | 4.86E-02 |
| ENSCAFG00000014924 | *MYOC* | -5.434488744 | 6.61E-04 | 4.86E-02 |
| ENSCAFG00000030902 | *TRIM59* | 1.264135843 | 6.61E-04 | 4.86E-02 |
| ENSCAFG00000019440 | *MECP2* | -0.767921111 | 6.63E-04 | 4.86E-02 |
| ENSCAFG00000030087 |  | 2.973130881 | 6.65E-04 | 4.86E-02 |
| ENSCAFG00000006162 | *SMAD9* | 0.798065077 | 6.67E-04 | 4.86E-02 |
| ENSCAFG00000020228 | *DHX38* | -0.568740556 | 6.71E-04 | 4.86E-02 |
| ENSCAFG00000003875 | *PLK4* | 2.881650357 | 6.72E-04 | 4.86E-02 |
| ENSCAFG00000013495 | *OLR1* | 3.747433434 | 6.72E-04 | 4.86E-02 |
| ENSCAFG00000007306 | *MAPRE1* | 1.062858751 | 6.75E-04 | 4.86E-02 |
| ENSCAFG00000009391 | *PTPN5* | 5.844480640 | 6.80E-04 | 4.88E-02 |
| ENSCAFG00000012717 | *QSOX1* | 1.337477246 | 6.86E-04 | 4.88E-02 |
| ENSCAFG00000031178 | *CNIH1* | 0.979393573 | 6.92E-04 | 4.88E-02 |
| ENSCAFG00000016479 | *TMEM184A* | -3.131257073 | 6.92E-04 | 4.88E-02 |
| ENSCAFG00000031992 | *SAR1B* | 0.814297204 | 6.97E-04 | 4.88E-02 |
| ENSCAFG00000020290 | *DAB2IP* | -1.067275241 | 7.00E-04 | 4.88E-02 |
| ENSCAFG00000013160 | *NPL* | -1.362785474 | 7.05E-04 | 4.88E-02 |
| ENSCAFG00000015037 | *DCUN1D5* | 0.889160835 | 7.05E-04 | 4.88E-02 |
| ENSCAFG00000032183 | *SGPP1* | 0.523551290 | 7.11E-04 | 4.89E-02 |
