# Supplementary material for: Biologically Enhanced Genome-Wide Association Study Provides Further Evidence for Candidate Loci and Discovers Novel Loci That Influence Risk of Anterior Cruciate Ligament Rupture in a Dog Model
Source: Front Genet. 2021 Mar 5;12:593515. doi: 10.3389/fgene.2021.593515 (PMC7982834; doi:10.3389/fgene.2021.593515)
Supplement: Supplementary file 5 [file Table_3.DOCX]

**Supplementary Table S3**. Differentially expressed genes identified through RNA sequencing of synovium tissue collected from dogs affected and unaffected with anterior cruciate ligament rupture.

| **Ensembl ID** | **Gene Name** | **logFC** | ***P*** | **Adjusted *P*** |
| --- | --- | --- | --- | --- |
| ENSCAFG00000009569 | *SPP1* | 6.213698843 | 5.56E-07 | 3.79E-03 |
| ENSCAFG00000012598 | *SLAMF7* | 3.671933543 | 8.99E-07 | 3.79E-03 |
| ENSCAFG00000012614 | *KCNK2* | 4.586032792 | 9.90E-07 | 3.79E-03 |
| ENSCAFG00000030867 | *HPCAL4* | 3.531466567 | 1.11E-06 | 3.79E-03 |
| ENSCAFG00000014432 |  | 7.073379802 | 2.34E-06 | 5.75E-03 |
| ENSCAFG00000016883 | *ITGAX* | 2.848214280 | 2.52E-06 | 5.75E-03 |
| ENSCAFG00000028568 | *JCHAIN* | 6.631592776 | 3.56E-06 | 6.11E-03 |
| ENSCAFG00000003757 | *OSTM1* | 0.991227213 | 4.38E-06 | 6.11E-03 |
| ENSCAFG00000016556 | *AQP9* | 3.464606398 | 4.40E-06 | 6.11E-03 |
| ENSCAFG00000017342 | *GPR65* | 3.025758701 | 4.75E-06 | 6.11E-03 |
| ENSCAFG00000011698 | *TLR7* | 2.847801092 | 4.91E-06 | 6.11E-03 |
| ENSCAFG00000012330 | *TMEM206* | 1.225133256 | 5.45E-06 | 6.22E-03 |
| ENSCAFG00000002660 |  | 2.966687022 | 6.12E-06 | 6.40E-03 |
| ENSCAFG00000013504 | *CLEC7A* | 4.133138932 | 6.65E-06 | 6.40E-03 |
| ENSCAFG00000031806 |  | 7.911716282 | 7.02E-06 | 6.40E-03 |
| ENSCAFG00000015017 | *ATP6V0A1* | 1.214559362 | 7.53E-06 | 6.40E-03 |
| ENSCAFG00000031273 |  | 5.354536483 | 8.00E-06 | 6.40E-03 |
| ENSCAFG00000013940 | *CLEC4E* | 3.212744124 | 8.42E-06 | 6.40E-03 |
| ENSCAFG00000010608 | *CFAP58* | 6.199051557 | 9.28E-06 | 6.40E-03 |
| ENSCAFG00000017616 | *LARP6* | -2.502719134 | 9.45E-06 | 6.40E-03 |
| ENSCAFG00000029357 | *NCEH1* | 1.120173547 | 9.81E-06 | 6.40E-03 |
| ENSCAFG00000005685 | *PAK5* | 5.795350091 | 1.14E-05 | 7.04E-03 |
| ENSCAFG00000031204 | *RGS10* | 2.514888716 | 1.24E-05 | 7.04E-03 |
| ENSCAFG00000031013 | *SAMSN1* | 3.032346558 | 1.24E-05 | 7.04E-03 |
| ENSCAFG00000002061 | *CD72* | 3.505614240 | 1.29E-05 | 7.04E-03 |
| ENSCAFG00000008408 | *TPD52* | 1.659460400 | 1.38E-05 | 7.28E-03 |
| ENSCAFG00000013495 | *OLR1* | 6.019565158 | 1.49E-05 | 7.55E-03 |
| ENSCAFG00000001246 | *PTCH1* | -1.831673152 | 1.78E-05 | 8.70E-03 |
| ENSCAFG00000013673 | *CXHXorf21* | 2.331615606 | 2.18E-05 | 1.02E-02 |
| ENSCAFG00000028603 |  | 3.163340197 | 2.23E-05 | 1.02E-02 |
| ENSCAFG00000012671 | *TRIM63* | 2.098492778 | 2.36E-05 | 1.04E-02 |
| ENSCAFG00000011041 | *CSF2RA* | 2.341655865 | 3.38E-05 | 1.28E-02 |
| ENSCAFG00000002515 | *MACC1* | 4.405367725 | 3.46E-05 | 1.28E-02 |
| ENSCAFG00000031786 |  | 8.711951968 | 3.47E-05 | 1.28E-02 |
| ENSCAFG00000006175 | *CDC25B* | -2.142961031 | 3.53E-05 | 1.28E-02 |
| ENSCAFG00000028667 | *CBLN3* | 2.236913637 | 3.65E-05 | 1.28E-02 |
| ENSCAFG00000017692 | *GLA* | 1.054104266 | 3.70E-05 | 1.28E-02 |
| ENSCAFG00000028509 |  | 7.174385771 | 3.83E-05 | 1.28E-02 |
| ENSCAFG00000013763 | *LTF* | 2.594059393 | 3.91E-05 | 1.28E-02 |
| ENSCAFG00000008026 |  | 2.579873726 | 3.96E-05 | 1.28E-02 |
| ENSCAFG00000004406 | *FBLN2* | -2.792105657 | 4.03E-05 | 1.28E-02 |
| ENSCAFG00000006691 | *SLC46A3* | 1.691301109 | 4.17E-05 | 1.28E-02 |
| ENSCAFG00000004595 |  | 5.140371591 | 4.30E-05 | 1.28E-02 |
| ENSCAFG00000032078 |  | 8.737113956 | 4.30E-05 | 1.28E-02 |
| ENSCAFG00000031733 |  | 7.230974876 | 4.33E-05 | 1.28E-02 |
| ENSCAFG00000007012 | *SPIC* | 6.183153325 | 4.45E-05 | 1.28E-02 |
| ENSCAFG00000004265 | *THNSL1* | 1.655225448 | 4.57E-05 | 1.28E-02 |
| ENSCAFG00000012206 | *ACKR3* | -2.722790918 | 4.57E-05 | 1.28E-02 |
| ENSCAFG00000000281 | *ABRACL* | 1.201527108 | 4.57E-05 | 1.28E-02 |
| ENSCAFG00000031201 |  | 2.923961378 | 5.96E-05 | 1.62E-02 |
| ENSCAFG00000015593 |  | 2.678330827 | 6.09E-05 | 1.62E-02 |
| ENSCAFG00000010997 | *CD80* | 2.092379802 | 6.25E-05 | 1.62E-02 |
| ENSCAFG00000031397 |  | 3.250093129 | 6.28E-05 | 1.62E-02 |
| ENSCAFG00000003945 | *DENND2A* | -1.557794039 | 6.64E-05 | 1.65E-02 |
| ENSCAFG00000002318 | *ARL4A* | -1.856899059 | 6.82E-05 | 1.65E-02 |
| ENSCAFG00000018482 | *CERS4* | -1.770924313 | 6.86E-05 | 1.65E-02 |
| ENSCAFG00000028762 | *KLRG1* | 2.992506318 | 6.92E-05 | 1.65E-02 |
| ENSCAFG00000013314 | *NCF2* | 2.183350391 | 7.18E-05 | 1.65E-02 |
| ENSCAFG00000007203 | *GGH* | 1.654501638 | 7.21E-05 | 1.65E-02 |
| ENSCAFG00000029297 | *ANKRD22* | 3.638889183 | 7.29E-05 | 1.65E-02 |
| ENSCAFG00000010711 | *ATP6V1A* | 1.484354604 | 7.40E-05 | 1.65E-02 |
| ENSCAFG00000001027 | *FAM91A1* | 1.100024849 | 7.55E-05 | 1.65E-02 |
| ENSCAFG00000012286 | *STRADB* | 1.403366871 | 7.70E-05 | 1.65E-02 |
| ENSCAFG00000030196 | *DRAM2* | 1.320128888 | 7.73E-05 | 1.65E-02 |
| ENSCAFG00000009101 | *ZHX3* | -1.177659593 | 7.93E-05 | 1.66E-02 |
| ENSCAFG00000001502 | *CARD10* | -2.144674886 | 8.03E-05 | 1.66E-02 |
| ENSCAFG00000003694 | *PRDM1* | 2.732698177 | 8.16E-05 | 1.66E-02 |
| ENSCAFG00000028847 |  | 6.327953766 | 8.24E-05 | 1.66E-02 |
| ENSCAFG00000015726 | *KMO* | 1.933268978 | 8.36E-05 | 1.66E-02 |
| ENSCAFG00000011188 | *GALNT3* | 2.566406481 | 8.79E-05 | 1.72E-02 |
| ENSCAFG00000023924 | *CD151* | -0.935065824 | 9.19E-05 | 1.73E-02 |
| ENSCAFG00000003662 | *TBC1D9* | 1.827712756 | 9.23E-05 | 1.73E-02 |
| ENSCAFG00000029467 |  | 7.178086654 | 9.24E-05 | 1.73E-02 |
| ENSCAFG00000001591 | *TREM1* | 2.888787101 | 9.68E-05 | 1.77E-02 |
| ENSCAFG00000013325 | *PHEX* | -4.294824759 | 9.72E-05 | 1.77E-02 |
| ENSCAFG00000002935 | *VRK2* | 0.819577208 | 9.92E-05 | 1.79E-02 |
| ENSCAFG00000029252 | *RND3* | -1.604965962 | 1.05E-04 | 1.87E-02 |
| ENSCAFG00000003859 |  | -3.251139406 | 1.11E-04 | 1.96E-02 |
| ENSCAFG00000012903 | *RUNX3* | 2.149604373 | 1.13E-04 | 1.97E-02 |
| ENSCAFG00000032751 | *CLEC2B* | 2.987859098 | 1.15E-04 | 1.97E-02 |
| ENSCAFG00000012883 | *ARHGDIB* | 1.340153868 | 1.17E-04 | 1.97E-02 |
| ENSCAFG00000014074 | *CRYAB* | -2.004377773 | 1.20E-04 | 1.97E-02 |
| ENSCAFG00000012569 | *CD84* | 2.327042299 | 1.20E-04 | 1.97E-02 |
| ENSCAFG00000038896 |  | 2.749665480 | 1.23E-04 | 1.97E-02 |
| ENSCAFG00000017192 | *HMMR* | 1.270231437 | 1.23E-04 | 1.97E-02 |
| ENSCAFG00000030258 | *IGHM* | 7.435020427 | 1.25E-04 | 1.97E-02 |
| ENSCAFG00000016245 |  | 2.845557434 | 1.27E-04 | 1.97E-02 |
| ENSCAFG00000013478 | *EDEM3* | 1.263358563 | 1.29E-04 | 1.97E-02 |
| ENSCAFG00000000419 | *AKAP12* | -2.408411931 | 1.31E-04 | 1.97E-02 |
| ENSCAFG00000002171 | *SYK* | 1.698324508 | 1.31E-04 | 1.97E-02 |
| ENSCAFG00000010491 | *LPCAT1* | 1.503404334 | 1.32E-04 | 1.97E-02 |
| ENSCAFG00000014799 |  | -1.501886779 | 1.32E-04 | 1.97E-02 |
| ENSCAFG00000025025 | *TRIM6* | 1.671293334 | 1.34E-04 | 1.98E-02 |
| ENSCAFG00000018465 | *PARP8* | 0.997311184 | 1.38E-04 | 2.00E-02 |
| ENSCAFG00000005038 | *MAP3K19* | 6.363575755 | 1.39E-04 | 2.00E-02 |
| ENSCAFG00000029125 | *SIRPB2* | 2.294016598 | 1.43E-04 | 2.03E-02 |
| ENSCAFG00000025113 | *CLEC12A* | 2.859536357 | 1.44E-04 | 2.03E-02 |
| ENSCAFG00000010198 | *RAB7B* | 1.333157193 | 1.48E-04 | 2.06E-02 |
| ENSCAFG00000031529 | *CLEC2D* | 4.991378001 | 1.49E-04 | 2.06E-02 |
| ENSCAFG00000032474 |  | 3.459286360 | 1.52E-04 | 2.07E-02 |
| ENSCAFG00000001712 | *LRRC19* | 2.655672911 | 1.53E-04 | 2.07E-02 |
| ENSCAFG00000009906 | *VSIG10* | -1.013423393 | 1.59E-04 | 2.13E-02 |
| ENSCAFG00000014349 | *TBC1D14* | 0.820903131 | 1.61E-04 | 2.14E-02 |
| ENSCAFG00000031853 |  | 6.533256849 | 1.66E-04 | 2.18E-02 |
| ENSCAFG00000020345 | *PTGFR* | 1.989957165 | 1.71E-04 | 2.19E-02 |
| ENSCAFG00000014879 | *LRRC25* | 2.361388721 | 1.71E-04 | 2.19E-02 |
| ENSCAFG00000016114 | *ADA2* | 2.169768782 | 1.74E-04 | 2.19E-02 |
| ENSCAFG00000010095 | *ATP6V1B2* | 1.006204618 | 1.75E-04 | 2.19E-02 |
| ENSCAFG00000001932 | *CNTFR* | -3.126359708 | 1.75E-04 | 2.19E-02 |
| ENSCAFG00000024111 |  | 6.351106634 | 1.76E-04 | 2.19E-02 |
| ENSCAFG00000001816 | *ATP8A1* | 1.345746061 | 1.78E-04 | 2.19E-02 |
| ENSCAFG00000004944 | *PCDH9* | 3.547100238 | 1.79E-04 | 2.19E-02 |
| ENSCAFG00000004854 | *ATP6V0B* | 1.115852038 | 1.83E-04 | 2.19E-02 |
| ENSCAFG00000032057 |  | 7.252134433 | 1.84E-04 | 2.19E-02 |
| ENSCAFG00000015176 |  | 6.721131662 | 1.84E-04 | 2.19E-02 |
| ENSCAFG00000020290 | *DAB2IP* | -1.210249499 | 1.87E-04 | 2.19E-02 |
| ENSCAFG00000006200 | *PLXNC1* | 2.740794473 | 1.87E-04 | 2.19E-02 |
| ENSCAFG00000007307 |  | -4.158353586 | 1.90E-04 | 2.20E-02 |
| ENSCAFG00000032358 |  | 9.302051606 | 1.94E-04 | 2.20E-02 |
| ENSCAFG00000009721 | *ALCAM* | 1.544928122 | 1.95E-04 | 2.20E-02 |
| ENSCAFG00000016584 | *HK3* | 3.973427471 | 1.95E-04 | 2.20E-02 |
| ENSCAFG00000029367 | *COL28A1* | -7.108851229 | 1.96E-04 | 2.20E-02 |
| ENSCAFG00000015441 | *LRMDA* | 1.516775601 | 1.99E-04 | 2.22E-02 |
| ENSCAFG00000000647 | *SERAC1* | 1.009119435 | 2.05E-04 | 2.24E-02 |
| ENSCAFG00000000398 | *LRP11* | 1.035341500 | 2.06E-04 | 2.24E-02 |
| ENSCAFG00000002323 | *MGAT4A* | 1.627362102 | 2.07E-04 | 2.24E-02 |
| ENSCAFG00000029821 | *IFNE* | 4.900089889 | 2.08E-04 | 2.24E-02 |
| ENSCAFG00000028917 | *SNX10* | 1.914326555 | 2.10E-04 | 2.25E-02 |
| ENSCAFG00000025192 | *CASP8* | 1.283884770 | 2.12E-04 | 2.26E-02 |
| ENSCAFG00000012621 | *LY9* | 2.059033234 | 2.20E-04 | 2.32E-02 |
| ENSCAFG00000011677 | *EPB41* | 1.154487787 | 2.27E-04 | 2.35E-02 |
| ENSCAFG00000023369 |  | 6.695293095 | 2.31E-04 | 2.35E-02 |
| ENSCAFG00000000100 | *SEC11C* | 1.340915129 | 2.33E-04 | 2.35E-02 |
| ENSCAFG00000000804 | *FBLN1* | -2.339621938 | 2.35E-04 | 2.35E-02 |
| ENSCAFG00000030838 | *PTGES* | -2.230404183 | 2.35E-04 | 2.35E-02 |
| ENSCAFG00000018852 | *TNFRSF17* | 4.481479689 | 2.38E-04 | 2.35E-02 |
| ENSCAFG00000009883 | *TENT5C* | 2.400632866 | 2.38E-04 | 2.35E-02 |
| ENSCAFG00000007617 | *ST8SIA4* | 2.647811526 | 2.38E-04 | 2.35E-02 |
| ENSCAFG00000000159 | *NEMP1* | 0.941952875 | 2.39E-04 | 2.35E-02 |
| ENSCAFG00000000931 | *NUS1* | 0.809258502 | 2.43E-04 | 2.37E-02 |
| ENSCAFG00000018540 | *MYO1F* | 1.997604391 | 2.46E-04 | 2.37E-02 |
| ENSCAFG00000032195 |  | 8.502290486 | 2.47E-04 | 2.37E-02 |
| ENSCAFG00000002918 | *RUFY3* | 0.858826968 | 2.48E-04 | 2.37E-02 |
| ENSCAFG00000009056 | *ADAM28* | 2.985633950 | 2.49E-04 | 2.37E-02 |
| ENSCAFG00000011458 | *LRMP* | 1.807997812 | 2.55E-04 | 2.38E-02 |
| ENSCAFG00000018492 | *CRLF3* | 1.021977310 | 2.57E-04 | 2.38E-02 |
| ENSCAFG00000009829 | *CD2* | 3.453788939 | 2.59E-04 | 2.38E-02 |
| ENSCAFG00000015000 | *TACC3* | 1.535507489 | 2.59E-04 | 2.38E-02 |
| ENSCAFG00000005586 | *AP3M2* | 1.708612865 | 2.63E-04 | 2.38E-02 |
| ENSCAFG00000007248 | *GNPTAB* | 1.097038136 | 2.63E-04 | 2.38E-02 |
| ENSCAFG00000010582 | *BOC* | -1.501817831 | 2.68E-04 | 2.38E-02 |
| ENSCAFG00000001966 | *SH3RF3* | 1.782370601 | 2.68E-04 | 2.38E-02 |
| ENSCAFG00000031104 | *CX3CR1* | 2.704939383 | 2.68E-04 | 2.38E-02 |
| ENSCAFG00000013544 | *HSPB1* | -1.16336307 | 2.69E-04 | 2.38E-02 |
| ENSCAFG00000004762 | *SLC25A19* | 0.915199894 | 2.71E-04 | 2.38E-02 |
| ENSCAFG00000010413 | *TMEM54* | -1.852637699 | 2.71E-04 | 2.38E-02 |
| ENSCAFG00000003388 | *SH3D21* | -1.795234059 | 2.72E-04 | 2.38E-02 |
| ENSCAFG00000010821 | *TMTC1* | -1.94810964 | 2.79E-04 | 2.42E-02 |
| ENSCAFG00000007954 | *TNKS1BP1* | -1.453417186 | 2.83E-04 | 2.43E-02 |
| ENSCAFG00000015177 | *SELL* | 3.433430108 | 2.91E-04 | 2.47E-02 |
| ENSCAFG00000024646 | *ERAP2* | 2.063998482 | 2.93E-04 | 2.47E-02 |
| ENSCAFG00000001675 | *CDKN2A* | 3.379241308 | 2.93E-04 | 2.47E-02 |
| ENSCAFG00000030602 |  | 5.272179495 | 2.94E-04 | 2.47E-02 |
| ENSCAFG00000002774 | *ZNF462* | -1.451829208 | 2.98E-04 | 2.47E-02 |
| ENSCAFG00000006243 | *GNPDA1* | 1.319626777 | 3.01E-04 | 2.47E-02 |
| ENSCAFG00000013773 | *BCR* | -0.92466753 | 3.02E-04 | 2.47E-02 |
| ENSCAFG00000023972 |  | 1.761585127 | 3.02E-04 | 2.47E-02 |
| ENSCAFG00000004692 | *DCLRE1C* | 0.838857894 | 3.03E-04 | 2.47E-02 |
| ENSCAFG00000007893 | *RBP4* | -2.212294766 | 3.06E-04 | 2.48E-02 |
| ENSCAFG00000011086 | *SLC16A6* | 2.041481228 | 3.09E-04 | 2.48E-02 |
| ENSCAFG00000008123 | *TENM3* | 1.205120317 | 3.10E-04 | 2.48E-02 |
| ENSCAFG00000030180 | *IL18* | 1.774505844 | 3.24E-04 | 2.56E-02 |
| ENSCAFG00000002138 | *IL18R1* | 2.449359135 | 3.25E-04 | 2.56E-02 |
| ENSCAFG00000011879 | *MOSPD2* | 1.144568468 | 3.26E-04 | 2.56E-02 |
| ENSCAFG00000015489 | *SCG3* | 2.658549720 | 3.30E-04 | 2.58E-02 |
| ENSCAFG00000010241 | *PXN* | -0.694933654 | 3.32E-04 | 2.58E-02 |
| ENSCAFG00000011828 | *KLHL6* | 2.014021954 | 3.36E-04 | 2.60E-02 |
| ENSCAFG00000033004 |  | 2.195463823 | 3.41E-04 | 2.63E-02 |
| ENSCAFG00000004011 | *TBXAS1* | 1.905871844 | 3.47E-04 | 2.66E-02 |
| ENSCAFG00000000103 | *ZNF532* | -1.13516722 | 3.51E-04 | 2.66E-02 |
| ENSCAFG00000031753 |  | 6.454972430 | 3.51E-04 | 2.66E-02 |
| ENSCAFG00000029313 | *CLDN1* | -2.254564035 | 3.54E-04 | 2.66E-02 |
| ENSCAFG00000030902 | *TRIM59* | 1.470517183 | 3.55E-04 | 2.66E-02 |
| ENSCAFG00000016515 | *PEAR1* | -1.769244482 | 3.64E-04 | 2.69E-02 |
| ENSCAFG00000019060 | *ABR* | 0.860432453 | 3.64E-04 | 2.69E-02 |
| ENSCAFG00000010949 | *SLC37A2* | 2.720502222 | 3.67E-04 | 2.70E-02 |
| ENSCAFG00000031669 | *CNRIP1* | -1.686883442 | 3.70E-04 | 2.71E-02 |
| ENSCAFG00000002590 |  | 2.527738934 | 3.78E-04 | 2.74E-02 |
| ENSCAFG00000032325 |  | 9.184766128 | 3.79E-04 | 2.74E-02 |
| ENSCAFG00000009516 | *LY86* | 2.031288824 | 3.82E-04 | 2.75E-02 |
| ENSCAFG00000011177 | *SLC7A7* | 1.541660577 | 3.85E-04 | 2.76E-02 |
| ENSCAFG00000015914 | *PELP1* | -0.913705653 | 3.96E-04 | 2.82E-02 |
| ENSCAFG00000016864 | *ELMSAN1* | -1.109595934 | 3.97E-04 | 2.82E-02 |
| ENSCAFG00000004694 | *PTCH2* | -2.523753328 | 4.05E-04 | 2.86E-02 |
| ENSCAFG00000033303 |  | -1.348261088 | 4.10E-04 | 2.87E-02 |
| ENSCAFG00000011657 | *EAF2* | 3.641094216 | 4.13E-04 | 2.87E-02 |
| ENSCAFG00000028982 |  | -1.989659723 | 4.14E-04 | 2.87E-02 |
| ENSCAFG00000011950 | *SLC18A2* | -3.620140891 | 4.18E-04 | 2.87E-02 |
| ENSCAFG00000001361 | *APOBEC3Z3* | 1.724474755 | 4.18E-04 | 2.87E-02 |
| ENSCAFG00000001738 | *GRM8* | 5.054399100 | 4.18E-04 | 2.87E-02 |
| ENSCAFG00000014790 |  | 1.291436406 | 4.21E-04 | 2.87E-02 |
| ENSCAFG00000029700 | *LPAR6* | 1.165671054 | 4.24E-04 | 2.87E-02 |
| ENSCAFG00000014924 | *MYOC* | -6.300208962 | 4.26E-04 | 2.87E-02 |
| ENSCAFG00000014213 | *POU2AF1* | 6.757271994 | 4.27E-04 | 2.87E-02 |
| ENSCAFG00000018158 | *ZNF521* | -1.091048329 | 4.37E-04 | 2.92E-02 |
| ENSCAFG00000016603 | *ITGAL* | 2.370717796 | 4.40E-04 | 2.92E-02 |
| ENSCAFG00000000848 | *DLA-DMA* | 2.793791543 | 4.47E-04 | 2.94E-02 |
| ENSCAFG00000009847 | *PCIF1* | -0.740891032 | 4.47E-04 | 2.94E-02 |
| ENSCAFG00000002796 | *CCDC88A* | 1.055071618 | 4.49E-04 | 2.94E-02 |
| ENSCAFG00000006720 | *AMOTL2* | -1.691677432 | 4.50E-04 | 2.94E-02 |
| ENSCAFG00000002519 | *ITGB8* | 3.475364680 | 4.60E-04 | 2.94E-02 |
| ENSCAFG00000002528 | *TGFBR1* | 1.201935991 | 4.60E-04 | 2.94E-02 |
| ENSCAFG00000002834 |  | 1.999514452 | 4.64E-04 | 2.94E-02 |
| ENSCAFG00000017697 |  | -1.900240042 | 4.65E-04 | 2.94E-02 |
| ENSCAFG00000005334 | *GPR180* | 1.053472712 | 4.66E-04 | 2.94E-02 |
| ENSCAFG00000013806 | *M6PR* | 0.978347582 | 4.68E-04 | 2.94E-02 |
| ENSCAFG00000009802 | *CYLD* | 0.945614844 | 4.68E-04 | 2.94E-02 |
| ENSCAFG00000020300 | *LPAR3* | 3.706304549 | 4.68E-04 | 2.94E-02 |
| ENSCAFG00000005613 | *SLC4A7* | 1.134362291 | 4.72E-04 | 2.94E-02 |
| ENSCAFG00000011751 | *CD86* | 2.370986364 | 4.72E-04 | 2.94E-02 |
| ENSCAFG00000008456 |  | 0.845816910 | 4.75E-04 | 2.94E-02 |
| ENSCAFG00000007417 | *MAN2A1* | 1.165508178 | 4.87E-04 | 2.99E-02 |
| ENSCAFG00000013485 | *KLF7* | -1.150227552 | 4.87E-04 | 2.99E-02 |
| ENSCAFG00000000812 | *DLA-DQA1* | 4.132343700 | 4.90E-04 | 2.99E-02 |
| ENSCAFG00000032183 | *SGPP1* | 0.548888979 | 4.95E-04 | 3.00E-02 |
| ENSCAFG00000014095 | *DERL3* | 4.255267613 | 4.95E-04 | 3.00E-02 |
| ENSCAFG00000005608 | *EDEM1* | 1.096294587 | 4.99E-04 | 3.01E-02 |
| ENSCAFG00000011727 | *MILR1* | 1.918381860 | 5.03E-04 | 3.02E-02 |
| ENSCAFG00000015599 |  | 2.359014093 | 5.04E-04 | 3.02E-02 |
| ENSCAFG00000030412 | *BCL2L11* | 0.891243316 | 5.10E-04 | 3.04E-02 |
| ENSCAFG00000016173 | *THRA* | -1.076557184 | 5.14E-04 | 3.05E-02 |
| ENSCAFG00000013805 | *CCR1* | 1.981163756 | 5.17E-04 | 3.05E-02 |
| ENSCAFG00000010623 | *SORCS3* | 4.304894070 | 5.28E-04 | 3.10E-02 |
| ENSCAFG00000010910 | *FEZ1* | -2.705216709 | 5.31E-04 | 3.10E-02 |
| ENSCAFG00000004491 | *LCP1* | 1.957337720 | 5.31E-04 | 3.10E-02 |
| ENSCAFG00000018711 | *RANBP3L* | 2.124913867 | 5.33E-04 | 3.10E-02 |
| ENSCAFG00000012205 | *INPP5F* | 0.898687940 | 5.49E-04 | 3.16E-02 |
| ENSCAFG00000000649 | *ATP6V1C1* | 0.803412846 | 5.51E-04 | 3.16E-02 |
| ENSCAFG00000032590 | *IL21R* | 4.050064604 | 5.51E-04 | 3.16E-02 |
| ENSCAFG00000001093 | *FAM49B* | 1.263853967 | 5.53E-04 | 3.16E-02 |
| ENSCAFG00000001786 | *ACO1* | 0.875965116 | 5.60E-04 | 3.18E-02 |
| ENSCAFG00000003452 | *KLHL32* | 2.778430796 | 5.82E-04 | 3.29E-02 |
| ENSCAFG00000013317 | *LMX1A* | -3.740683651 | 5.90E-04 | 3.31E-02 |
| ENSCAFG00000003397 | *TFEC* | 2.512629099 | 5.95E-04 | 3.31E-02 |
| ENSCAFG00000032313 | *FGL1* | -4.09879967 | 5.95E-04 | 3.31E-02 |
| ENSCAFG00000008959 | *RIPK2* | 0.774117837 | 5.96E-04 | 3.31E-02 |
| ENSCAFG00000007693 | *LPXN* | 1.832647088 | 5.96E-04 | 3.31E-02 |
| ENSCAFG00000007882 | *PCSK1* | 2.674606864 | 6.21E-04 | 3.42E-02 |
| ENSCAFG00000000086 | *CDH20* | 8.406890367 | 6.21E-04 | 3.42E-02 |
| ENSCAFG00000003611 | *PRR12* | -0.955322493 | 6.24E-04 | 3.42E-02 |
| ENSCAFG00000006055 | *ATP2C1* | 0.664942989 | 6.29E-04 | 3.43E-02 |
| ENSCAFG00000007361 | *EPB41L4A* | -1.750559981 | 6.37E-04 | 3.46E-02 |
| ENSCAFG00000012410 | *CRTC3* | -0.914653146 | 6.42E-04 | 3.48E-02 |
| ENSCAFG00000005847 | *ADAM9* | 0.969598387 | 6.54E-04 | 3.53E-02 |
| ENSCAFG00000031415 |  | 6.933636703 | 6.59E-04 | 3.54E-02 |
| ENSCAFG00000031078 |  | 6.006381826 | 6.66E-04 | 3.56E-02 |
| ENSCAFG00000004284 | *LURAP1* | -1.838642803 | 6.75E-04 | 3.59E-02 |
| ENSCAFG00000004379 | *FNDC3A* | 0.813367824 | 6.77E-04 | 3.59E-02 |
| ENSCAFG00000008617 | *GLRB* | -1.797406204 | 6.79E-04 | 3.59E-02 |
| ENSCAFG00000005003 | *NCKAP5* | 2.763915999 | 6.88E-04 | 3.59E-02 |
| ENSCAFG00000011908 | *SHTN1* | 1.500444979 | 6.88E-04 | 3.59E-02 |
| ENSCAFG00000014564 |  | 3.310855789 | 6.88E-04 | 3.59E-02 |
| ENSCAFG00000010948 | *PREX1* | 1.357161169 | 6.90E-04 | 3.59E-02 |
| ENSCAFG00000029632 |  | 4.821331743 | 6.96E-04 | 3.61E-02 |
| ENSCAFG00000013924 | *PLCD3* | -1.208003979 | 7.00E-04 | 3.61E-02 |
| ENSCAFG00000005354 | *KLF6* | -1.880448476 | 7.02E-04 | 3.61E-02 |
| ENSCAFG00000015584 |  | 5.234599930 | 7.07E-04 | 3.62E-02 |
| ENSCAFG00000018672 | *VTN* | -3.573501745 | 7.14E-04 | 3.64E-02 |
| ENSCAFG00000018546 | *NNT* | 1.021580536 | 7.15E-04 | 3.64E-02 |
| ENSCAFG00000005465 | *DZIP1* | -0.95979865 | 7.17E-04 | 3.64E-02 |
| ENSCAFG00000039260 |  | -1.918793875 | 7.21E-04 | 3.64E-02 |
| ENSCAFG00000017735 | *GSKIP* | 1.006033769 | 7.29E-04 | 3.67E-02 |
| ENSCAFG00000009364 | *TFG* | 2.102334946 | 7.42E-04 | 3.71E-02 |
| ENSCAFG00000001028 | *RNF217* | 1.116101839 | 7.43E-04 | 3.71E-02 |
| ENSCAFG00000006668 | *KBTBD8* | 1.248902907 | 7.48E-04 | 3.73E-02 |
| ENSCAFG00000004316 | *GSAP* | 1.176339521 | 7.51E-04 | 3.73E-02 |
| ENSCAFG00000003626 | *IL15* | 0.962650314 | 7.66E-04 | 3.79E-02 |
| ENSCAFG00000005155 | *MFSD11* | 0.931126767 | 7.73E-04 | 3.79E-02 |
| ENSCAFG00000010536 | *CARMIL1* | -1.495286372 | 7.78E-04 | 3.79E-02 |
| ENSCAFG00000006249 | *NDFIP1* | 0.852280495 | 7.80E-04 | 3.79E-02 |
| ENSCAFG00000018209 | *EPN2* | -1.169322669 | 7.85E-04 | 3.79E-02 |
| ENSCAFG00000007590 | *ZNF507* | -0.768255287 | 7.86E-04 | 3.79E-02 |
| ENSCAFG00000000741 | *SYBU* | -1.76832498 | 7.86E-04 | 3.79E-02 |
| ENSCAFG00000010550 | *ZBTB8OS* | 0.644458470 | 7.87E-04 | 3.79E-02 |
| ENSCAFG00000009255 | *PTPN22* | 2.555657364 | 7.88E-04 | 3.79E-02 |
| ENSCAFG00000008310 | *ZFHX4* | -2.015368428 | 7.94E-04 | 3.80E-02 |
| ENSCAFG00000012787 |  | -2.63524956 | 7.97E-04 | 3.80E-02 |
| ENSCAFG00000025145 | *ZNF777* | -0.945788633 | 7.98E-04 | 3.80E-02 |
| ENSCAFG00000009257 | *MED13L* | -0.805663293 | 8.02E-04 | 3.80E-02 |
| ENSCAFG00000008669 | *TMEM144* | 1.811930465 | 8.07E-04 | 3.81E-02 |
| ENSCAFG00000000480 | *NIPAL2* | 2.155259815 | 8.18E-04 | 3.83E-02 |
| ENSCAFG00000032359 | *GIMAP2* | 1.674323773 | 8.19E-04 | 3.83E-02 |
| ENSCAFG00000007755 | *MAT2A* | 0.905125684 | 8.19E-04 | 3.83E-02 |
| ENSCAFG00000008833 | *ACP2* | 0.680472397 | 8.28E-04 | 3.86E-02 |
| ENSCAFG00000009379 | *CRY2* | -1.212960736 | 8.36E-04 | 3.87E-02 |
| ENSCAFG00000000814 |  | 3.950721966 | 8.39E-04 | 3.87E-02 |
| ENSCAFG00000013961 | *TP63* | 2.887275122 | 8.40E-04 | 3.87E-02 |
| ENSCAFG00000016746 | *FAM155B* | -2.059335542 | 8.47E-04 | 3.89E-02 |
| ENSCAFG00000029121 | *FAM78A* | 1.392007847 | 8.50E-04 | 3.89E-02 |
| ENSCAFG00000023704 | *SLAMF6* | 4.279752763 | 8.62E-04 | 3.94E-02 |
| ENSCAFG00000017626 | *FAXDC2* | -0.769473352 | 8.76E-04 | 3.96E-02 |
| ENSCAFG00000032102 | *DLA-DMB* | 2.685522565 | 8.78E-04 | 3.96E-02 |
| ENSCAFG00000032369 | *ZDHHC22* | 1.137467124 | 8.79E-04 | 3.96E-02 |
| ENSCAFG00000034404 |  | 1.870436481 | 8.79E-04 | 3.96E-02 |
| ENSCAFG00000009498 | *ADA* | -1.345181833 | 8.86E-04 | 3.97E-02 |
| ENSCAFG00000008347 | *NUGGC* | 3.199922659 | 8.88E-04 | 3.97E-02 |
| ENSCAFG00000006047 | *LETM2* | -0.88837382 | 8.93E-04 | 3.97E-02 |
| ENSCAFG00000002576 | *HIVEP3* | 1.602055106 | 8.93E-04 | 3.97E-02 |
| ENSCAFG00000023843 |  | 8.945197701 | 9.00E-04 | 3.99E-02 |
| ENSCAFG00000009152 | *HDAC7* | -1.413010818 | 9.15E-04 | 4.04E-02 |
| ENSCAFG00000015482 | *TOP3B* | -0.81971959 | 9.17E-04 | 4.04E-02 |
| ENSCAFG00000028636 | *IGFBP6* | -2.825321894 | 9.25E-04 | 4.06E-02 |
| ENSCAFG00000024010 | *TLR1* | 1.566012233 | 9.33E-04 | 4.08E-02 |
| ENSCAFG00000009036 | *PLCB2* | 1.803494924 | 9.37E-04 | 4.09E-02 |
| ENSCAFG00000007579 | *PIK3CB* | 0.958859642 | 9.43E-04 | 4.10E-02 |
| ENSCAFG00000030297 | *GRB2* | 0.867612766 | 9.51E-04 | 4.11E-02 |
| ENSCAFG00000003685 | *CREM* | 1.049570764 | 9.54E-04 | 4.11E-02 |
| ENSCAFG00000005999 | *NHLRC3* | 1.148953215 | 9.54E-04 | 4.11E-02 |
| ENSCAFG00000008351 | *TLR2* | 1.529756728 | 9.61E-04 | 4.13E-02 |
| ENSCAFG00000017181 | *MAT2B* | 0.973740676 | 9.68E-04 | 4.14E-02 |
| ENSCAFG00000018703 | *SASH3* | 1.721082385 | 9.70E-04 | 4.14E-02 |
| ENSCAFG00000019766 | *CEPT1* | 0.776628264 | 9.73E-04 | 4.14E-02 |
| ENSCAFG00000009407 | *LPCAT2* | 1.756714080 | 9.86E-04 | 4.18E-02 |
| ENSCAFG00000011334 | *RCC1L* | -0.778257461 | 9.89E-04 | 4.18E-02 |
| ENSCAFG00000001480 | *TRIOBP* | -0.975666623 | 1.00E-03 | 4.22E-02 |
| ENSCAFG00000008520 | *GPR171* | 3.185886802 | 1.01E-03 | 4.24E-02 |
| ENSCAFG00000012014 | *SH3BP4* | -1.336205269 | 1.02E-03 | 4.25E-02 |
| ENSCAFG00000013191 | *NRP2* | 1.182057627 | 1.02E-03 | 4.25E-02 |
| ENSCAFG00000017712 | *IGSF6* | 2.005602154 | 1.02E-03 | 4.25E-02 |
| ENSCAFG00000002762 | *EML6* | 2.209226310 | 1.03E-03 | 4.25E-02 |
| ENSCAFG00000003709 | *CD37* | 1.618904538 | 1.03E-03 | 4.25E-02 |
| ENSCAFG00000012934 | *MAP3K3* | -0.695822133 | 1.03E-03 | 4.25E-02 |
| ENSCAFG00000030892 | *PERP* | -3.465986664 | 1.03E-03 | 4.25E-02 |
| ENSCAFG00000030284 |  | 7.746740090 | 1.04E-03 | 4.27E-02 |
| ENSCAFG00000007143 | *ASAH1* | 1.143954981 | 1.05E-03 | 4.27E-02 |
| ENSCAFG00000003663 | *GSN* | -1.96568204 | 1.05E-03 | 4.27E-02 |
| ENSCAFG00000014222 | *IFT80* | 0.686198223 | 1.05E-03 | 4.27E-02 |
| ENSCAFG00000002233 | *NPR2* | -0.812039114 | 1.06E-03 | 4.28E-02 |
| ENSCAFG00000006830 | *MSR1* | 2.199623954 | 1.06E-03 | 4.28E-02 |
| ENSCAFG00000011077 | *ADARB1* | -1.016605532 | 1.07E-03 | 4.29E-02 |
| ENSCAFG00000011353 | *DENND1B* | 1.112480125 | 1.07E-03 | 4.29E-02 |
| ENSCAFG00000014295 | *SMC4* | 1.009362538 | 1.08E-03 | 4.31E-02 |
| ENSCAFG00000014164 | *MDGA2* | 4.543286536 | 1.08E-03 | 4.31E-02 |
| ENSCAFG00000002420 | *MEOX2* | -1.673905672 | 1.09E-03 | 4.34E-02 |
| ENSCAFG00000000157 | *DCC* | 3.488890293 | 1.10E-03 | 4.34E-02 |
| ENSCAFG00000018169 | *CCL14* | -2.888577651 | 1.10E-03 | 4.34E-02 |
| ENSCAFG00000018857 | *GPC4* | -1.504300283 | 1.10E-03 | 4.34E-02 |
| ENSCAFG00000029541 | *ATF5* | -1.069695439 | 1.10E-03 | 4.34E-02 |
| ENSCAFG00000006150 | *PANK2* | 0.858186447 | 1.11E-03 | 4.34E-02 |
| ENSCAFG00000010003 | *MOB2* | -1.01718631 | 1.11E-03 | 4.34E-02 |
| ENSCAFG00000032319 |  | 3.165475904 | 1.12E-03 | 4.36E-02 |
| ENSCAFG00000016689 | *CLTB* | -0.905903874 | 1.14E-03 | 4.36E-02 |
| ENSCAFG00000011782 | *GFRA1* | 2.559935085 | 1.14E-03 | 4.36E-02 |
| ENSCAFG00000040020 |  | 4.424728541 | 1.14E-03 | 4.36E-02 |
| ENSCAFG00000013697 | *C17H1orf162* | 1.990612908 | 1.15E-03 | 4.36E-02 |
| ENSCAFG00000032328 |  | 7.943324878 | 1.15E-03 | 4.36E-02 |
| ENSCAFG00000011286 | *OSTC* | 0.766024958 | 1.15E-03 | 4.36E-02 |
| ENSCAFG00000003855 | *LDAH* | 0.620361687 | 1.15E-03 | 4.36E-02 |
| ENSCAFG00000020411 | *NEGR1* | 4.740391257 | 1.15E-03 | 4.36E-02 |
| ENSCAFG00000014255 |  | -1.088579211 | 1.15E-03 | 4.36E-02 |
| ENSCAFG00000009615 | *STK4* | 0.774839996 | 1.15E-03 | 4.36E-02 |
| ENSCAFG00000012556 | *HTRA1* | 1.984362820 | 1.15E-03 | 4.36E-02 |
| ENSCAFG00000012489 | *CASTOR1* | -1.477718249 | 1.16E-03 | 4.36E-02 |
| ENSCAFG00000008477 | *BLNK* | 1.425747712 | 1.16E-03 | 4.37E-02 |
| ENSCAFG00000020251 | *NOB1* | -0.710541181 | 1.18E-03 | 4.41E-02 |
| ENSCAFG00000002131 | *ERMP1* | 1.029399516 | 1.19E-03 | 4.43E-02 |
| ENSCAFG00000005210 | *IL2RA* | 2.676936303 | 1.19E-03 | 4.43E-02 |
| ENSCAFG00000003252 | *AOAH* | 2.665948188 | 1.19E-03 | 4.43E-02 |
| ENSCAFG00000033317 |  | 4.065095303 | 1.20E-03 | 4.44E-02 |
| ENSCAFG00000011474 | *FCMR* | 3.061666908 | 1.20E-03 | 4.45E-02 |
| ENSCAFG00000005109 | *CXCR4* | 2.978250701 | 1.21E-03 | 4.47E-02 |
| ENSCAFG00000004355 | *LIMS2* | -1.443418543 | 1.21E-03 | 4.47E-02 |
| ENSCAFG00000031795 |  | 5.862947677 | 1.22E-03 | 4.48E-02 |
| ENSCAFG00000010684 | *LINS1* | 0.717028060 | 1.22E-03 | 4.48E-02 |
| ENSCAFG00000037363 |  | 2.601006832 | 1.24E-03 | 4.52E-02 |
| ENSCAFG00000010157 | *BACE2* | -0.747302629 | 1.24E-03 | 4.52E-02 |
| ENSCAFG00000000876 | *PARVG* | 1.910027110 | 1.25E-03 | 4.53E-02 |
| ENSCAFG00000013278 |  | -2.011793109 | 1.25E-03 | 4.53E-02 |
| ENSCAFG00000017315 | *SEL1L* | 1.042945265 | 1.25E-03 | 4.53E-02 |
| ENSCAFG00000007034 | *LYN* | 1.216517139 | 1.27E-03 | 4.56E-02 |
| ENSCAFG00000002999 | *ZMPSTE24* | 0.616130990 | 1.27E-03 | 4.56E-02 |
| ENSCAFG00000004615 | *APOC1* | 3.256107273 | 1.27E-03 | 4.56E-02 |
| ENSCAFG00000006909 | *LMO1* | 1.436784793 | 1.30E-03 | 4.65E-02 |
| ENSCAFG00000010177 | *KDM1B* | 0.759674389 | 1.31E-03 | 4.66E-02 |
| ENSCAFG00000029740 | *MSRB3* | -0.956781659 | 1.31E-03 | 4.66E-02 |
| ENSCAFG00000013774 | *MAP3K14* | -1.082900598 | 1.32E-03 | 4.66E-02 |
| ENSCAFG00000017259 | *MAGT1* | 0.819404867 | 1.32E-03 | 4.66E-02 |
| ENSCAFG00000029752 | *C11H5orf15* | 0.880069263 | 1.32E-03 | 4.66E-02 |
| ENSCAFG00000023802 | *ARMCX1* | -1.162969916 | 1.33E-03 | 4.66E-02 |
| ENSCAFG00000007503 | *SBNO1* | 0.757325106 | 1.33E-03 | 4.66E-02 |
| ENSCAFG00000003198 | *FKBP15* | 0.654482450 | 1.34E-03 | 4.68E-02 |
| ENSCAFG00000003004 | *PPT1* | 1.140460162 | 1.34E-03 | 4.68E-02 |
| ENSCAFG00000029976 | *DNAJB9* | 1.162154252 | 1.35E-03 | 4.69E-02 |
| ENSCAFG00000024944 |  | 3.163963024 | 1.35E-03 | 4.69E-02 |
| ENSCAFG00000029534 | *BLOC1S2* | 0.668503758 | 1.36E-03 | 4.69E-02 |
| ENSCAFG00000009506 | *ABI3BP* | 2.689304831 | 1.36E-03 | 4.69E-02 |
| ENSCAFG00000003056 | *PLEKHA8* | 1.148424698 | 1.36E-03 | 4.69E-02 |
| ENSCAFG00000031724 | *CACNG5* | -2.831629768 | 1.37E-03 | 4.70E-02 |
| ENSCAFG00000014175 | *ITGA4* | 2.015414652 | 1.37E-03 | 4.70E-02 |
| ENSCAFG00000004887 |  | -1.384185562 | 1.37E-03 | 4.70E-02 |
| ENSCAFG00000031832 | *CCDC115* | 0.635666577 | 1.39E-03 | 4.75E-02 |
| ENSCAFG00000024087 | *CGAS* | 2.002637282 | 1.40E-03 | 4.75E-02 |
| ENSCAFG00000002941 | *ME1* | 0.756090318 | 1.40E-03 | 4.75E-02 |
| ENSCAFG00000013782 | *CCRL2* | 1.914764347 | 1.40E-03 | 4.75E-02 |
| ENSCAFG00000032614 |  | 2.204900771 | 1.41E-03 | 4.75E-02 |
| ENSCAFG00000012780 | *CD3G* | 2.776625264 | 1.41E-03 | 4.75E-02 |
| ENSCAFG00000031403 |  | 7.628902642 | 1.41E-03 | 4.75E-02 |
| ENSCAFG00000005419 | *PNP* | -0.86489843 | 1.42E-03 | 4.76E-02 |
| ENSCAFG00000034279 |  | -1.54729037 | 1.43E-03 | 4.78E-02 |
| ENSCAFG00000008401 | *P2RX7* | 1.397050037 | 1.44E-03 | 4.78E-02 |
| ENSCAFG00000012781 | *XPR1* | 0.732539865 | 1.44E-03 | 4.78E-02 |
| ENSCAFG00000030935 |  | 8.623120221 | 1.44E-03 | 4.78E-02 |
| ENSCAFG00000009592 | *NELL2* | 5.727098265 | 1.44E-03 | 4.78E-02 |
| ENSCAFG00000031101 | *CD48* | 1.655372263 | 1.45E-03 | 4.79E-02 |
| ENSCAFG00000012892 | *ERP27* | 3.061820381 | 1.46E-03 | 4.79E-02 |
| ENSCAFG00000018063 | *ACSL4* | 0.985470788 | 1.46E-03 | 4.79E-02 |
| ENSCAFG00000008279 | *PTPRJ* | 1.573381321 | 1.46E-03 | 4.79E-02 |
| ENSCAFG00000003184 | *CEP68* | -1.088652083 | 1.49E-03 | 4.82E-02 |
| ENSCAFG00000006546 | *ARID5A* | -1.054388677 | 1.49E-03 | 4.82E-02 |
| ENSCAFG00000012789 | *PTPRO* | 1.505502333 | 1.49E-03 | 4.82E-02 |
| ENSCAFG00000011099 | *AMZ2* | 1.034499541 | 1.50E-03 | 4.82E-02 |
| ENSCAFG00000012293 | *ADCY4* | -1.426991836 | 1.50E-03 | 4.82E-02 |
| ENSCAFG00000031177 | *ST6GALNAC6* | -0.954971176 | 1.51E-03 | 4.82E-02 |
| ENSCAFG00000003383 | *ADAM17* | 0.950596829 | 1.51E-03 | 4.82E-02 |
| ENSCAFG00000011395 | *NCF1* | 1.675863852 | 1.51E-03 | 4.82E-02 |
| ENSCAFG00000030001 |  | 6.875948787 | 1.52E-03 | 4.82E-02 |
| ENSCAFG00000009912 | *ERG* | -1.162903042 | 1.52E-03 | 4.82E-02 |
| ENSCAFG00000001215 | *GFRA3* | -1.926455064 | 1.52E-03 | 4.82E-02 |
| ENSCAFG00000009129 |  | 0.890292581 | 1.52E-03 | 4.82E-02 |
| ENSCAFG00000019698 | *PALM* | -2.489277872 | 1.53E-03 | 4.82E-02 |
| ENSCAFG00000015182 | *GLB1L* | 1.349657740 | 1.53E-03 | 4.82E-02 |
| ENSCAFG00000007167 | *CHD7* | 0.797599143 | 1.53E-03 | 4.82E-02 |
| ENSCAFG00000015982 | *CDC42BPA* | -0.866542301 | 1.53E-03 | 4.82E-02 |
| ENSCAFG00000005738 | *SLC30A6* | 0.690589889 | 1.53E-03 | 4.82E-02 |
| ENSCAFG00000031478 |  | 0.798736773 | 1.54E-03 | 4.82E-02 |
| ENSCAFG00000017669 | *BTK* | 1.539784611 | 1.54E-03 | 4.82E-02 |
| ENSCAFG00000010603 | *RGS1* | 2.147254304 | 1.54E-03 | 4.82E-02 |
| ENSCAFG00000031968 | *C5H11orf52* | 2.111748866 | 1.54E-03 | 4.82E-02 |
| ENSCAFG00000014603 | *TRIM9* | 2.862697852 | 1.55E-03 | 4.82E-02 |
| ENSCAFG00000012086 | *CTSS* | 2.149518683 | 1.57E-03 | 4.87E-02 |
| ENSCAFG00000011265 | *PTPRC* | 1.815104855 | 1.59E-03 | 4.95E-02 |
| ENSCAFG00000006502 | *TPP1* | 1.550571291 | 1.60E-03 | 4.95E-02 |
| ENSCAFG00000016231 | *FUT8* | 1.238204511 | 1.61E-03 | 4.99E-02 |
